# Engineering Lung-Inspired Flow Field Geometries for Electrochemical Flow Cells with Stereolithography 3D Printing

**DOI:** 10.1021/acssuschemeng.3c00848

**Published:** 2023-07-24

**Authors:** Vanesa Muñoz-Perales, Maxime van der Heijden, Pablo A. García-Salaberri, Marcos Vera, Antoni Forner-Cuenca

**Affiliations:** † Department of Thermal and Fluids Engineering, Universidad Carlos III de Madrid, 28911 Leganés, Spain; ‡ Electrochemical Materials and Systems, Department of Chemical Engineering and Chemistry, Eindhoven University of Technology, Eindhoven 5600 MB, The Netherlands

**Keywords:** fractal geometries, lung-inspired design, flow reactor, aqueous redox flow batteries, high performance, homogeneous electrolyte distribution, electrochemical diagnostics, numerical simulation

## Abstract

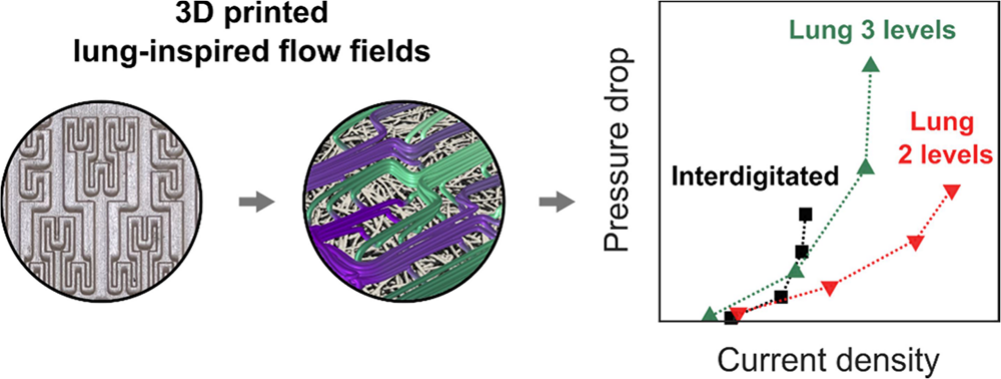

Electrochemical flow reactors are increasingly relevant platforms in emerging sustainable energy conversion and storage technologies. As a prominent example, redox flow batteries, a well-suited technology for large energy storage if the costs can be significantly reduced, leverage electrochemical reactors as power converting units. Within the reactor, the flow field geometry determines the electrolyte pumping power required, mass transport rates, and overall cell performance. However, current designs are inspired by fuel cell technologies but have not been engineered for redox flow battery applications, where liquid-phase electrochemistry is sustained. Here, we leverage stereolithography 3D printing to manufacture lung-inspired flow field geometries and compare their performance to conventional flow field designs. A versatile two-step process based on stereolithography 3D printing followed by a coating procedure to form a conductive structure is developed to manufacture lung-inspired flow field geometries. We employ a suite of fluid dynamics, electrochemical diagnostics, and finite element simulations to correlate the flow field geometry with performance in symmetric flow cells. We find that the lung-inspired structural pattern homogenizes the reactant distribution throughout the porous electrode and improves the electrolyte accessibility to the electrode reaction area. In addition, the results reveal that these novel flow field geometries can outperform conventional interdigitated flow field designs, as these patterns exhibit a more favorable balance of electrical and pumping power, achieving superior current densities at lower pressure loss. Although at its nascent stage, additive manufacturing offers a versatile design space for manufacturing engineered flow field geometries for advanced flow reactors in emerging electrochemical energy storage technologies.

## Abstract 2

Lung-inspired flow fields improve flow battery performance which can facilitate the deployment of large-scale energy storage technologies to integrate renewables.

## Introduction

Stationary energy storage technologies are poised to play a pivotal role in the integration of renewable energies into the electricity grid.^1,2^ In this context, redox flow batteries (RFBs) are a well-suited solution for large-scale, long-duration, and low-cost energy storage.^3,4^ RFBs are rechargeable batteries where the electrochemical reactions occur in a flow reactor composed of stacked electrochemical cells. The active redox species dissolved in liquid electrolytes are stored in external tanks and pumped through the electrochemical stack by a hydraulic system to charge and discharge the battery. Their unique configuration enables the decoupling of power (size of the electrochemical stack) and energy (electrolyte volume stored in the tanks).^5−7^ Despite their technological advances, RFBs have witnessed limited market penetration due to a number of technical and economic challenges. To date, researchers have aimed to improve the performance of the system by designing new electrolytes formulations,^8,9^ improved electrodes and membranes,^10,11^ and engineered electrochemical reactor designs.^12,13^

**Figure 1:**
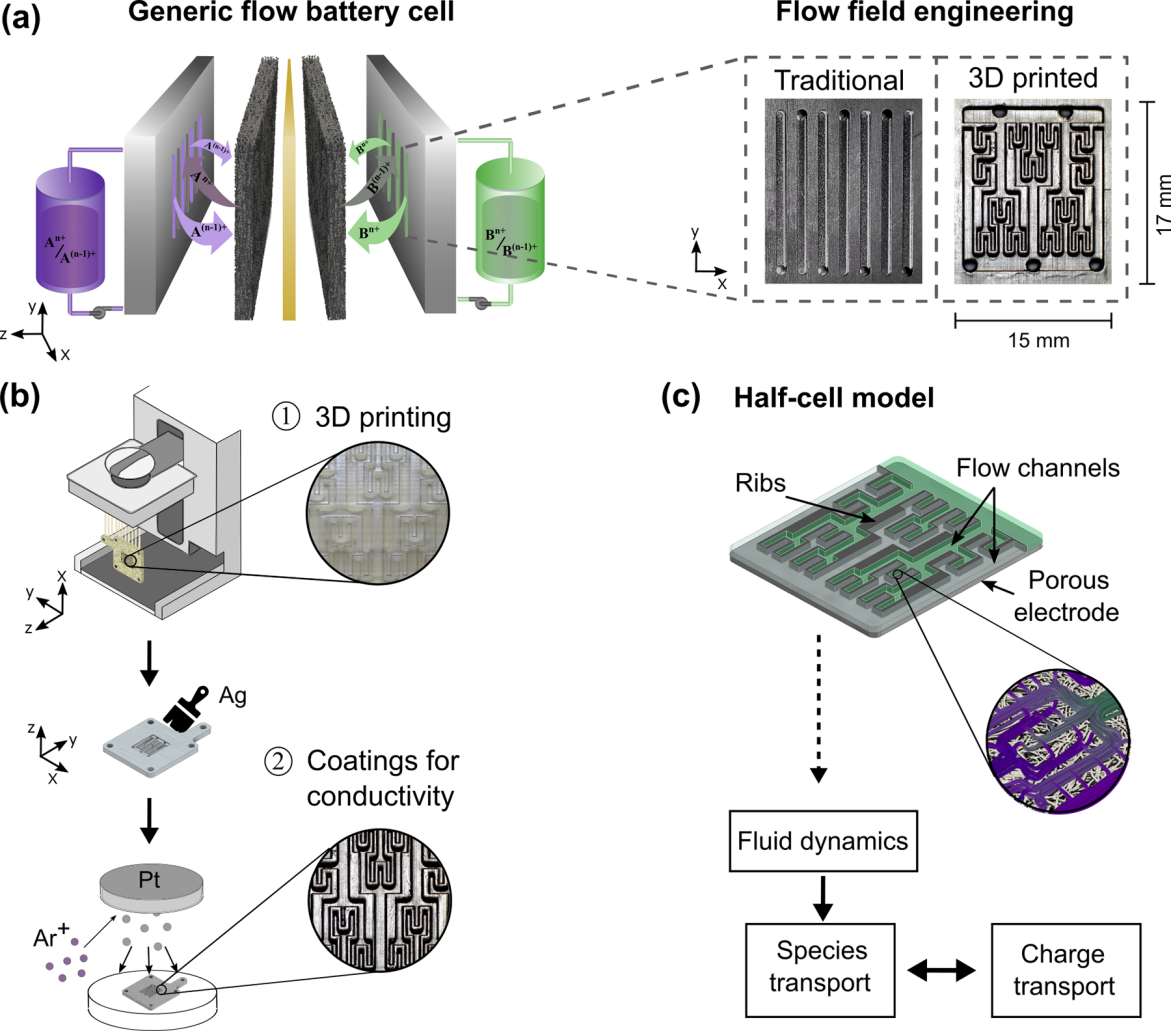
Concept for 3D printed lung-inspired flow fields integrated into a redox flow battery cell. (a) Illustration of a single flow cell of a generic RFB. Inset view: microscopic pictures of the traditional graphite interdigitated flow field (left) and 3D printed lung-inspired flow field (right). (b) Illustration of the two-step manufacturing process of the 3D printed flow fields. (c) Schematic of the continuum 3D half-cell model.

Within the electrochemical cell, the flow fields (Figure 1a), generally integrated within the current collectors or bipolar plates, are performance-defining components that govern the electrochemical performance and overall energy efficiency through pressure losses.^14^ The flow field geometry determines the electrolyte distribution into the porous electrode, the electrode compression, the accessibility of the active electrode surface area, the system pressure drop, and the electronic contact resistances.^15,16^ Drawing inspiration from polymer electrolyte fuel cells, current RFB technologies leverage flow-through, interdigitated, and serpentine flow field designs.^17,18^ However, while functional, these designs have not been tailored for the specific requirements of RFBs where liquid reactive flows are sustained. Recently, innovative flow field geometries featuring hierarchical and fractal patterns, which provide more uniform reactant distribution in electrochemical cells, have been studied. Trogadas et al. proposed a lung-inspired approach in three dimensions of the flow field to improve reactant distribution in polymer electrolyte fuel cells.^19^ An improvement of 20–30% in electrochemical performance was demonstrated in comparison with serpentine flow fields and a 50–75% lower pressure drop. Zeng et al. demonstrated the potential of hierarchical interdigitated designs to minimize pumping losses and increase voltage efficiency in RFBs.^20^ The authors proposed a two-level interdigitated flow field in three dimensions that enhances the mass transport in the electrode and decreases the hydraulic resistances under the ribs of the flow field.

In parallel, topology optimization and machine learning have emerged as a powerful toolset to engineer material geometries using a bottom-up approach.^21,22^ Recently, this non-heuristic method has been applied to design flow fields in electrochemical reactors.^23−25^ Yaji et al. formulated a topology optimization process for the flow field in an all-vanadium RFB as a maximization problem for the generation rate of vanadium species resulting in an improved electrochemical reaction in the porous electrode.^26^ They found interdigitated patterns with disconnected branched channels to be an optimized configuration for permeable porous media. Behrou et al. also demonstrated the effectivity of using density-based topology optimization for the design of flow fields in proton exchange membrane fuel cells by maximizing the power generation and the homogeneity of the current density distribution.^27^ The obtained flow field geometries presented similarities with bio-inspired patterns (e.g., lung-inspired) that improve the reactant distribution and provide a better trade-off between higher power generation and reduced pressure losses. Beck et al. used computational three-dimensional optimization to design micro-architected 3D porous electrodes with variable porosity which lead to higher power efficiencies in RFBs.^28^ Based on this work, Lin et al. used a similar methodology to design 3D flow fields in all-vanadium RFBs with full three-dimensional geometry variation,^29^ which resulted in significantly reduced power losses by evolving 3D geometries from the standard interdigitated flow field to optimized patterns in the three spatial dimensions. In an alternative effort, Wan et al. used machine learning methods to identify eight potential flow field designs which can achieve superior limiting currents (with an increase of 22%), resulting in improvements up to 11% of the battery energy efficiency compared with the serpentine flow field.^30^ In both the non-heuristic and heuristic studies,^19,20^ the potential and relevance of fractal and hierarchical features in flow fields were identified and exploided. However, these studies have been developed numerically, and their findings have only been experimentally tested by few groups. Wan et al. used manufacturing methods based on machining the channels on the graphite plates,^30^ whereas Trogadas et al. manufactured their lung-inspired flow fields for polymer electrolyte fuel cells using 3D printing via direct metal laser sintering, achieving the defined geometry with a resolution of 33 μm.^19^

Previous research leveraged interdigitated flow fields as the base pattern for optimization studies.^20,26,29^ However, in our previous work, we demonstrated the interdependence between the electrode microstructure and the flow field geometry.^31^ We showed that when using non-woven carbon paper electrodes, interdigitated flow fields are an effective combination to distribute the electrolyte. Moreover, interdigitated designs with a high number of channels, thus providing a larger electrolyte exchange perimeter, and wide rib patterns provide an optimal trade-off between the electrical and pumping power. These findings help to explain why the previously mentioned studies on topology optimization found hierarchical interdigitated flow field designs to be optimal in terms of electrochemical performance and pressure drop. In this context, the main challenge remains to introduce complex flow field geometries tailored for a specific electrode microstructure and to implement them in flow batteries.

The complexity of the previously proposed geometries might challenge the economic viability of the manufacturing process. In recent years, three-dimensional (3D) printing, as an additive manufacturing technique, has been increasingly deployed due to its decreased costs and versatile design possibilities.^32^ A few previous studies have used 3D printing techniques to manufacture electrodes,^33,34^ flow fields,^19^ or other cell components such as endplates^35,36^ and frames.^37^ However, the application of 3D printing technologies for the fabrication of functional electrochemical components is still in an early stage where new 3D printing methodologies using functional conductive materials need to be developed and tested.^38−40^

Here, we present two novel 3D printed lung-inspired flow field geometries for advanced flow cell architectures in RFBs. We propose a rapid and flexible two-step manufacturing process based on stereolithography (SLA) 3D printing (see Figure 1b). Before manufacturing, the performance of the proposed designs was evaluated by a continuum 3D model of one half-cell. Once the designs were positively evaluated, they were manufactured and experimentally tested. First, we quantify their hydraulic resistance by pressure drop measurements and extract the apparent permeability values from Darcy–Forchheimer fittings. Second, we characterize their electrochemical performance at different electrolyte superficial velocities by polarization and electrochemical impedance spectroscopy measurements. Finally, we use the half-cell continuum 3D model (Figure 1c) to provide a comprehensive understanding of the velocity field distribution in the flow channels and porous electrode, as well as concentration, reaction source, and mass transfer 2D contours in the entire 3D porous domain. While this work focuses on new flow field geometries for carbon paper-based electrodes, we anticipate the proposed methodology can be applied to a broad range of electrochemical reactors.

## Methods

### Manufacturing of the Flow Fields

**Figure 2:**
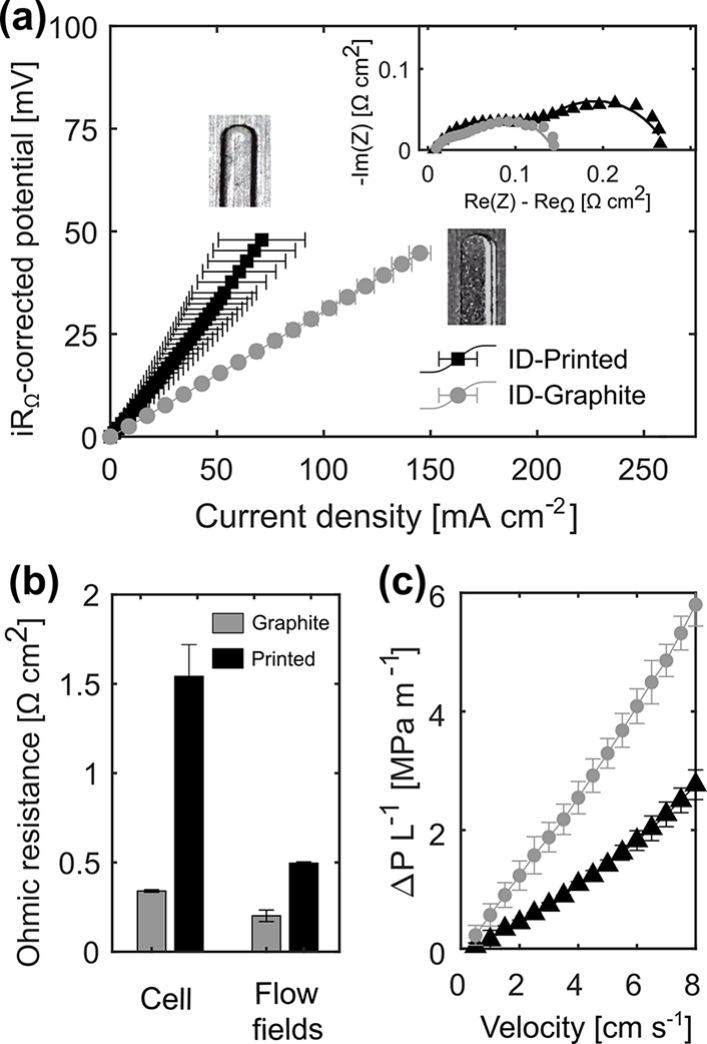
Comparison between the graphite ID flow field and the printed ID flow field. (a) *iR*_Ω_-corrected polarization at 3.5 cm s^–1^ electrolyte velocity using a 50% SOC electrolyte of Fe^2+^/Fe^3+^ in 2.0 M of HCl, together with the corresponding EIS subtracting the ohmic resistance. A microscopic image of the end part of one single channel in the graphite and printed flow fields is added to the figure. (b) Ohmic resistance of the electrodes normalized using their geometrical area and obtained from the EIS measurement under two different configurations: (i) regular cell with all components as in the electrochemical experiments (denominated “Cell”) and (ii) empty cell (no electrolyte, electrodes, or membrane) with both flow fields touching each other (denominated “Flow fields”). (c) Pressure drop per unit length at different electrolyte velocities in the electrode.

**Figure 3:**
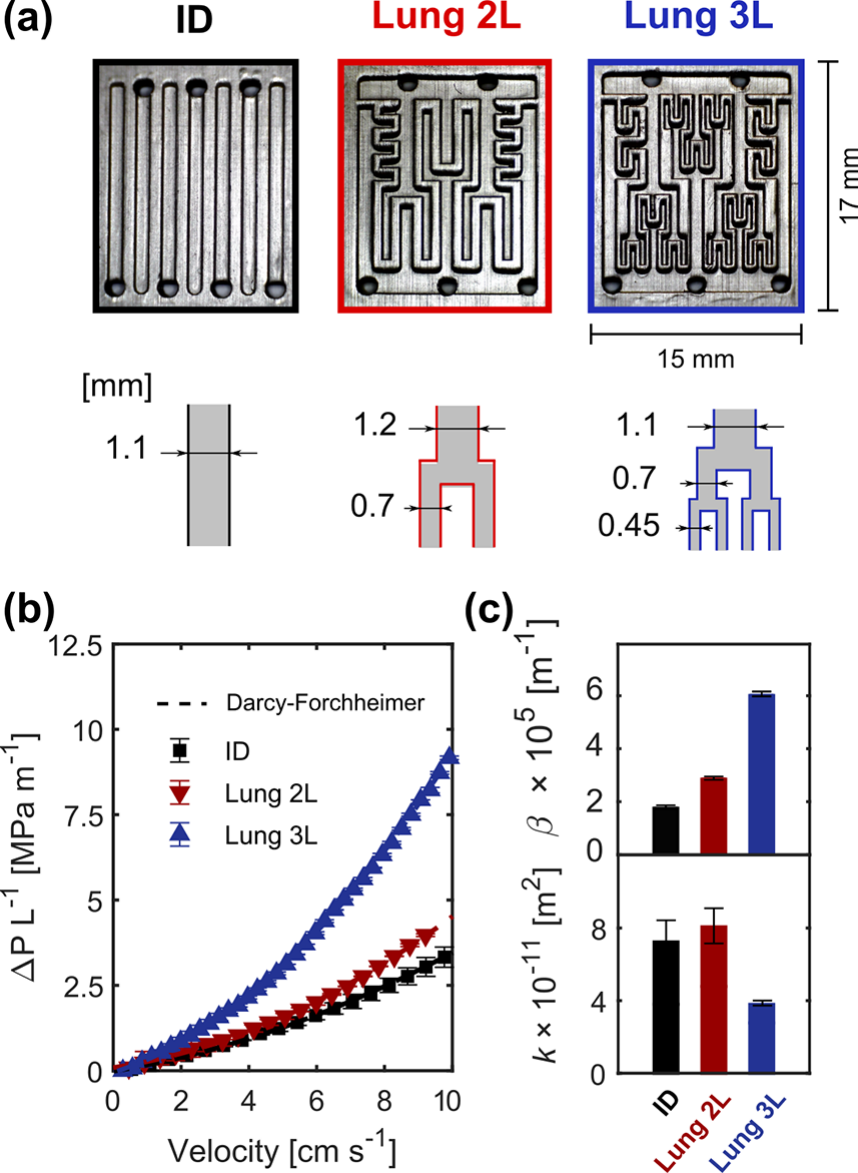
Images of the 3D printed flow fields and hydraulic characterization. (a) Optical microscopic images of the ID-baseline, Lung 2L, and Lung 3L printed flow fields and a schematic representation of the key cannel dimensions. (b) Pressure drop measurements per unit length vs electrolyte velocity in the electrode of the printed electrode-flow field systems together with the Darcy–Forchheimer fitting. (c) Fitted hydraulic apparent permeability (bottom) and apparent Forchheimer coefficients (top) of the electrode-flow field systems.

A flexible manufacturing method consisting of two steps was developed for the fabrication of novel flow field geometries. First, the flow field geometries (40 × 40 × 3.2 mm^3^) were designed using Autodesk Inventor software (see CAD drawings in Figure S1). The designs were exported and loaded into the 3D printer PreForm (Formlabs) software, being translated to printable structures by adding pillars and a baseplate connected to the 3D design. A clear acrylate-based UV-curing High Temp V2 (Formlabs) resin was used in the SLA 3D printer. The SLA 3D printer was operated with a laser wavelength of 405 nm, a spot size of 85 μm, and a power of 120 mW. The resulting printing resolution in the *XY* plane was 25 μm and the laser spot size 85 μm. The total printing time was ∼10 h and multiple flow fields (e.g., from 1 to 10) could be printed at once without increasing the printing time. Later, the printed pieces were washed using the Form Wash (Formlabs) in isopropanol alcohol for 5 min to remove the excess resin. Then, a curing step was carried out in the Form Cure (Formlabs) for 120 min and 80 °C under air. Once the part was cured, a conductive silver paint (SPI supplies #05002-AB with bulk resistivity of 3 × 10^–5^ Ω cm) with a brush applicator cap was applied on the 3D printed surface and dried for 24 h at room temperature. Lastly, the silver-coated flow field samples were sputtered with platinum at 80 mA for 200 s in a JEOL, JFC-2300HR argon environment Sputter coater. Three different geometries were printed: the conventional interdigitated design used as baseline, and two new proposed geometries which will be denominated hereafter “Lung-inspired.” Their performance was previously evaluated against the conventional graphite milling (Figure 2). The resulting printed flow field geometries are presented in Figure 3a and their key dimensions are listed in Table 1.

**Table 1:** Geometrical Parameters of ID, Lung 2L, and Lung 3L, with a Channel Depth of 0.5 mm^a^

dimension	ID	Lung 2L	Lung 3L
main channel width [mm]	1.1	1.2	1.1
2nd level channel width [mm]		0.7	0.7
3rd level channel width [mm]			0.45
exchange perimeter (*P*_E_) [mm]	96.0	113.1	183.9
channel-contact area [mm^2^]	115.3	154.9	177.7

a The CAD drawings and calculation of exchange perimeter is provided in Section S1 (Figures S1 and S2).

### Pressure Drop Measurements

The pressure drop through the carbon paper electrode in combination with the flow field designs was measured in a custom flow cell setup (see Figure S3). The device is identical as the electrochemical cell (Figure S4), but the membrane was replaced by a solid aluminum plate to avoid the electrolyte passing through the membrane. To analyze the specific pressure drop of each flow field in combination with the carbon paper electrode, the fluid was pumped through the cell at different flow rates. The pressure was measured at the inlet and outlet positions of the cell using a digital pressure gauge (Stauff SPG-DIGI-USB) to calculate the pressure difference. For simplicity, water was used as a fluid, since it has similar density, viscosity, surface tension, and wetting properties as the aqueous electrolyte used for the electrochemical tests. To deconvolute the individual contributions of the flow field and the electrode to the total pressure drop, the cell pressure drop was also measured for an empty cell (i.e., without porous electrodes but using a gasket with the same thickness) for the different flow fields. The measurements were taken at different electrolyte velocities in the electrode within the range of 0.5–8 cm s^–1^. Finally, to obtain the hydraulic apparent permeability, the experimental data were fitted to the Darcy–Forchheimer equation^41^
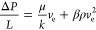
1where Δ*P* is the pressure drop (Pa), *L* is the electrode length (m), *v*_e_ is the electrolyte velocity in the electrode (m s^–1^), *μ* and *ρ* are the electrolyte viscosity (Pa s^–1^) and density (kg m^–3^), respectively, *k* is the hydraulic apparent permeability (*m*^2^), and *β* is the apparent Forchheimer coefficient (m^–1^).

### Electrochemical Characterization

A single-electrolyte flow cell configuration was used to evaluate the electrochemical performance of the 3D printed flow fields.^42,43^ A schematic of the 3D cell components is presented in Figure S4, consisting of two half-cells separated by a Nafion 212 membrane, each half-cell with a porous carbon paper electrode (Freudenberg H23, Fuel Cell Store, with an uncompressed thickness of 210 μm, thermally pre-treated at 450 °C for 12 h^44^ in a Nabertherm muffle oven model C290). The specific dimensions and drawings of the flow fields can be found in Figure S1. The studied flow field is stacked in the cell using incompressible Teflon gaskets (thickness of ∼170 μm) to ensure an electrode compression of 20%. During all experiments, the flow cell architecture remained the same and only the flow field was varied. The electrolyte, membrane, and electrodes were replaced by fresh ones when testing every different flow field and their corresponding repetitions. The electrolyte was pumped through LS-14 tubing to the symmetric cell using a Masterflex L/S Easy-LoadII pump.

The kinetically facile redox couple Fe^2+^/Fe^3+^ was selected as the active redox species for the electrolyte. A solution of 0.1 M ferrous chloride hydrate (FeCl_2_·4H2O, 98%, Sigma-Aldrich) and 0.1 M ferric chloride hydrate (FeCl_3_·6H_2_O, 97%, Sigma-Aldrich) in 2.0 M chlorhydric acid (37% w/w HCl from Sigma-Aldrich diluted in deionized water) was prepared for the electrochemical performance measurements. Limiting current density experiments were performed using a diluted solution of 0.6/3 mM Fe^2+^/Fe^3+^ in 2.0 M HCl.

The electrochemical experiments were performed in a sequential manner in the following order: (1) limiting current measurements, (2) electrochemical impedance spectroscopy (EIS), and (3) polarization measurements. For the limiting current measurements, the system was first purged and filled with the 0.6/3 mM electrolyte and then the pump was calibrated for all different flow fields. Then, the system was preconditioned for 30 min by pumping electrolyte at 5 cm s^–1^ through the cell and applying 25 mV voltage steps between 0 and 0.2 V, repeated several times until a stable current response was reached. Once the system was stabilized, the limiting current was measured with the polarization technique at different superficial velocities. For that, the flow rate was set according to the desired electrolyte velocity, which was calculated according to
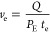
2where *Q* is the flow rate (m^3^ s^–1^), *t*_e_ is the electrode thickness (m), and *P*_E_ is the electrolyte exchange perimeter (m) calculated as the average perimeter of the inlet and outlet channel perimeter, excluding the outer cell boundaries (see Figure S2 and Table S1).

EIS and polarization were measured in a two steps sequence at every velocity after finishing the limiting current experiment. The system was purged first and filled then with 25 mL of the 0.1/0.1 M Fe^2+^/Fe^3+^ electrolyte. EIS was first measured at open circuit voltage, with an amplitude of 10 mV over a frequency range of 10 kHz to 30 mHz, 6 points per decade, 3 measurements per frequency, and a waiting time of 0.1 period before each frequency. The ohmic resistance of the studied cell was obtained from the high-frequency intercept which accounts for the cumulative electronic contact resistance and the membrane resistance in the cell. To deconvolute the charge and mass transfer resistances, an equivalent circuit model was used to fit the averaged experimental values of two repetitions of each experiment (see the ‘‘Results’’ section for further explanations). The resistance values are represented throughout the manuscript in a normalized basis using the geometrical area of the electrode (2.55 cm^2^). Secondly, polarization measurements were performed by potentiostatic holds of 1 min at constant cell voltage steps of 20 mV in a 0–0.6 V range and the steady-state current was recorded (one point per second). Due to the single-electrolyte flow cell configuration, the same reaction occurs in both half-cells but in opposite directions, resulting in a zero open circuit voltage. Thus, the applied potential corresponds to the overpotential of the cell (involving ohmic, kinetic, and transport resistances) and the *iR*_Ω_-corrected potential represented in the polarization curves refers to the potential applied to the cell subtracting the ohmic overpotential losses,^45^ representing only the charge and mass transfer overpotential losses. The last 20 points of the step were used and averaged. The impedance and polarization sequences were repeated at four different electrolyte velocities, 0.5, 1.5, 3.5, and 5 cm s^–1^. All experiments in this work were repeated twice. The reproducibility was previously verified using graphite-based interdigitated flow fields for seven cells with the same configuration (see Figure S5).

### Macroscopic Numerical 3D Simulations

To support the experimental results, numerical simulations of the 3D flow cell were carried out. To this goal, the flow domain was simplified to a half-cell exploiting the symmetry of the system. COMSOL Multiphysics was used for CFD simulations of the fluid dynamics coupled with the electrochemistry in the porous domain (see 3D geometry and mesh in Figure S6). Modeling of a half-compartment in single-electrolyte flow cells can be done accurately without including complex phenomena such as crossover thanks to the symmetric electrochemical configuration (same electrolyte in both half-cells).

The electrolyte flow in the flow channels and the electrode obeys the continuity equation for incompressible flow
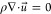
3along with the Navier–Stokes and Brinkman equations in the flow channels and the porous electrode respectively
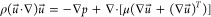
4
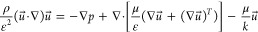
5where *u* is the local electrolyte velocity (m s^–1^), *p* is the fluid pressure (Pa), μ is the viscosity of the electrolyte (Pa s), and ε and *k* are the porosity (−) and permeability (m^2^) of the porous electrode, respectively. The model parameters can be found in Table 2.

**Table 2:** Numerical Values Adopted for the Parameters of the Numerical Model

symbol	quantity	value
*a* (ECSA)	electrochemical surface area [m^–1^]	1.314 × 10^4^^a^
*k*_L_	electrolyte conductivity [mS cm^–1^]	450^47^
σ_e_	electrode conductivity [mS cm^–1^]	3000^47^
ε	porosity	0.74^45^
*k*	permeability	2 × 10^–11^^45^
*k*^0^	reaction rate constant [m s^–1^]	1.1 × 10^–5^^48^
α_a_	anodic transfer coefficient [−]	0.5^49^
α_c_	cathodic transfer coefficient [−]	0.5^49^
ρ	electrolyte density [kg m^–3^]	1015^50^
Μ	electrolyte viscosity [Pa s]	1.143 × 10^–3^^51^
*D*_Fe^2+^_	diffusion coefficient of Fe^2+^ [m^2^ s^–1^]	5.7 × 10^–10^^52^
*D*_Fe^3+^_	diffusion coefficient of Fe^3+^ [m^2^ s^–1^]	4.8 × 10^–10^^52^
*E*_eq_	equilibrium potential [V]	0.771^6^
*R*_m_	membrane resistance [Ω]	0.0356^b^

a Measured experimentally (see Figures S9 and S10 in Section S5).

b Calculated (Section S4c).

The steady-state species conservation equation in the flow channels and the porous electrodes is the convection–diffusion-reaction Nernst–Planck equation
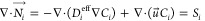
6where migration is neglected due to the excess of supporting electrolyte.^46^ In the above equation, *N_i_* is the molar flux vector (mol m^–2^ s^–1^), *S_i_* is the chemical source term (mol m^–3^ s^–1^), *C_i_* is the bulk concentration (mol m^–3^), and  is the effective diffusivity of species *i* (m^2^ s^–1^). The latter is computed using the Bruggeman correction as a function of the porosity, ε = 1 in the flow channels and ε < 1 in the porous electrode, and the species diffusivity *D_i_* in the fluid.

Charge conservation is applied to couple the ionic current density in the electrolyte, *i*_L_, with the electronic current density in the electrode, *i*_e_, both related to the local current density at the electrode surface
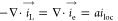
7where *a* is the electrochemical surface area of the electrode (m^2^ m^–3^) estimated by capacity measurements through cyclic voltammetry and the linear fit of the average capacity current at different scan rates using the graphite interdigitated flow field (see procedure in Section S5). The ionic and electronic currents are defined as
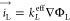
8
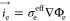
9where Φ_L_ and Φ_e_ are the liquid and solid potentials (V),  and  are their effective conductivities given by the Bruggeman correction (S m^–1^), and *i*_loc_ is the local current density (A m^–2^) defined by the Butler–Volmer equation
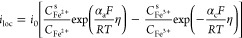
10where η = Φ_e_ – Φ_L_ – *E*_eq_ is the reaction overpotential (V), α_a_ and α_c_ are the anodic and cathodic charge transfer coefficients (−), and *i*_0_ is the exchange current density (A m^–2^) calculated as
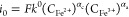
11where *k*^0^ is the reaction rate constant (m s^–1^). In the Butler–Volmer equation, the species concentrations at the electrode surface () are obtained assuming a linear diffusion layer from the bulk fluid to the electrode surface (see Section S4b). To this end, a local mass transfer coefficient (*k*_m_) depending on the velocity field is used in the model defined by^53^
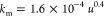
12

The above equations are complemented with the following boundary conditions. At the current collector interface Φ_e_ = V_cell_ and at the membrane interface Φ_L_ = ΔΦ_m_ = *R*_m_*I*_m_.^54^ The value of Φ_L_ at the membrane was assumed to be constant and equal to 5.5 mV for all applied voltages (see Section S4c). Electronic insulation is imposed at the membrane interface while ionic insulation is applied at the current collector interface. All remaining surfaces were assumed to be electrically insulated. The outlet pressure is set to *p*_out_ = *p*_atm_. A uniform electrolyte velocity is specified at the inlets of the channels calculated from the stipulated flow rate, *Q*. At the channel inlets, the concentration of species is imposed to be *C*_Fe^2+^,0_ and *C*_Fe^3+^,0_. For the remaining boundaries, the flux of species is set to zero. The mesh independence analysis can be found in Table S3 and Figure S7. The model validation is reported in Figure S8 for the three different flow fields at 3.5 cm s^–1^ of electrolyte velocity.

## Results and Discussion

### 3D Printed Geometries

Stereolithography 3D printing offers a powerful approach for the manufacturing of complex flow field geometries for RFBs. SLA is one of the earliest 3D printing methods,^55^ where an ultraviolet laser is used for photopolymerization of liquid resins, resulting in a polymerized 3D print. Since the flow field designs in this setup also work as current collectors, they must be made conductive in order to collect the current from the electrode. Therefore, a two-step method was adopted, as shown Figure 1b and described in detail in the methodology section. For industrialization, more economic and rapid techniques such as milling would be suitable to prepare these flow field designs at scale. However, the purpose of this study is to demonstrate that 3D printing coupled with conductive coatings is a versatile technique to manufacture flow field geometries unattainable with other manufacturing technologies.

To prove the feasibility of the proposed manufacturing process in Figure 2, we compare the resulting performance of the printed flow fields to that of the traditional graphite ones. The electrochemical performance of the cell is evaluated through the *iR*_Ω_-corrected potential to isolate activation and mass transfer potential losses. Polarization data reveals a significantly lower electrochemical performance for the printed flow field compared to the graphite milled flow plate (Figure 2a). We partially attribute the lower performance to a higher ohmic resistance of the 3D printed structure (Figure 2b) driven by the lower electric conductivity of the material which may lead to a distributed resistance^56,57^ as can be observed in the impedance plot in Figure 2a. To shed light on the electrical conductivity of both the graphite and printed flow fields, their associated ohmic resistance losses are compared in Figure 2b. In the bar plots, the values obtained from the regular cell with all the components are compared to those in an empty cell with only the flow fields stacked to each other (Figure S11a,b). The analysis on the ohmic resistance evinces the lower electrical conductivity of the printed flow field (128 ± 2 S m^–1^) compared to the graphite flow fields (318 ± 36 S m^–1^) (see Table S4 and Section S6 for the calculation details). When comparing the ohmic resistance difference in the regular cell with the isolated measurement of the flow fields in an empty cell, the resistance difference is found to be less significant. This supports the hypothesis of the existence of distributed resistance in the cell, contributing to the overall cell resistance and explaining the differences in the *iR*_Ω_-corrected potential measurements (Figure 2a). Furthermore, in Figure 2c, we analyzed the pressure drop, showing higher pressure losses for the printed flow field, which can be explained by deviations on the flow field morphology between the 3D printed designs and the milled graphite plates. Morphological differences are to be expected by, for example, gravity effects^58^ as after the curing process, the channel edges were round instead of sharp as in the graphite flow field. Additionally, the differences in roughness of the graphite and the 3D printed polymer can also be a factor contributing to the pressure loss differences.

Two new flow field geometries based on branched interdigitated geometries, tailored for carbon paper-based electrodes, were 3D printed: a lung-inspired flow field with two levels (Lung 2L) and three levels (Lung 3L) of branching. In Figure 3a, the optical microscopic pictures of the ID, Lung 2L, and Lung 3L printed geometries are presented. The new lung-based designs have three inlet channels (bottom) and two outlet channels (top). The targeted flow field structures were successfully printed according to the CAD design dimensions. However, gravity effects were observed during the curing process of the resin, resulting in smooth edges due to resin spreading, which could be beneficial for the flow distribution.

### Hydraulic Analysis

Flowing electrolyte through the electrochemical reactor requires a certain pumping power which affects the overall energy efficiency. Thus, we first evaluate the hydraulic behavior of every flow field with the carbon paper electrode by performing pressure drop measurements. In Figure 3b, the pressure drop normalized by the unit length of the flow reactor (i.e., electrode length) is shown at different electrolyte velocities together with the apparent permeability and Forchheimer coefficient (β) obtained from the Darcy–Forchheimer fittings (Table S5). The ID and the Lung 2L provide similar pressure losses (below 4 MPa m^–1^ at an electrolyte velocity of 10 cm s^–1^), while the Lung 3L results in significantly larger pressure drop providing 9 MPa m^–1^ for the same electrolyte velocity. These results can be explained by analyzing the hydraulic permeabilities obtained from the Darcy–Forchheimer fittings (Figure 3c), as the Lung 3L exhibits a significantly lower value of apparent permeability (4.01 × 10^–11^ m^2^) compared to the Lung 2L and ID (8.16 × 10^–11^ and 8.57 × 10^–11^ m^2^, respectively). To further investigate this, we analyze the apparent Forchheimer coefficients in Figure 3c, which quantifies the nonlinear effects in porous media (inertial effects).^59^ The differences in the geometry of the three flow fields have an impact on the microscopic inertial effects that distort the velocity and pressure fields.^60^ We hypothesize that due to the complex geometrical characteristics of the Lung 3L design and the higher local electrolyte velocities, this design results in the largest apparent Forchheimer coefficient. Additionally, the deconvolution of the pressure drop contributions by the flow field and the electrode, as shown in Table S6, reveals that the number of channels and complexity in the geometry of the flow fields contributes significantly on decreasing the hydraulic apparent permeability. The Lung 3L flow field contains one additional level of smaller channels compared to the Lung 2L, providing the largest exchange perimeter and channel-contact area (see Table 1). On the contrary, the Lung 3L features the highest contribution to the total pressure drop (73%), while ID and Lung 2L contribute less (53 and 59%, respectively). Therefore, designs with a greater number and levels of branches can hamper the overall efficiency of the electrochemical stack due to increased hydraulic requirements to maintain the desired electrolyte flow condition.

**Figure 4:**
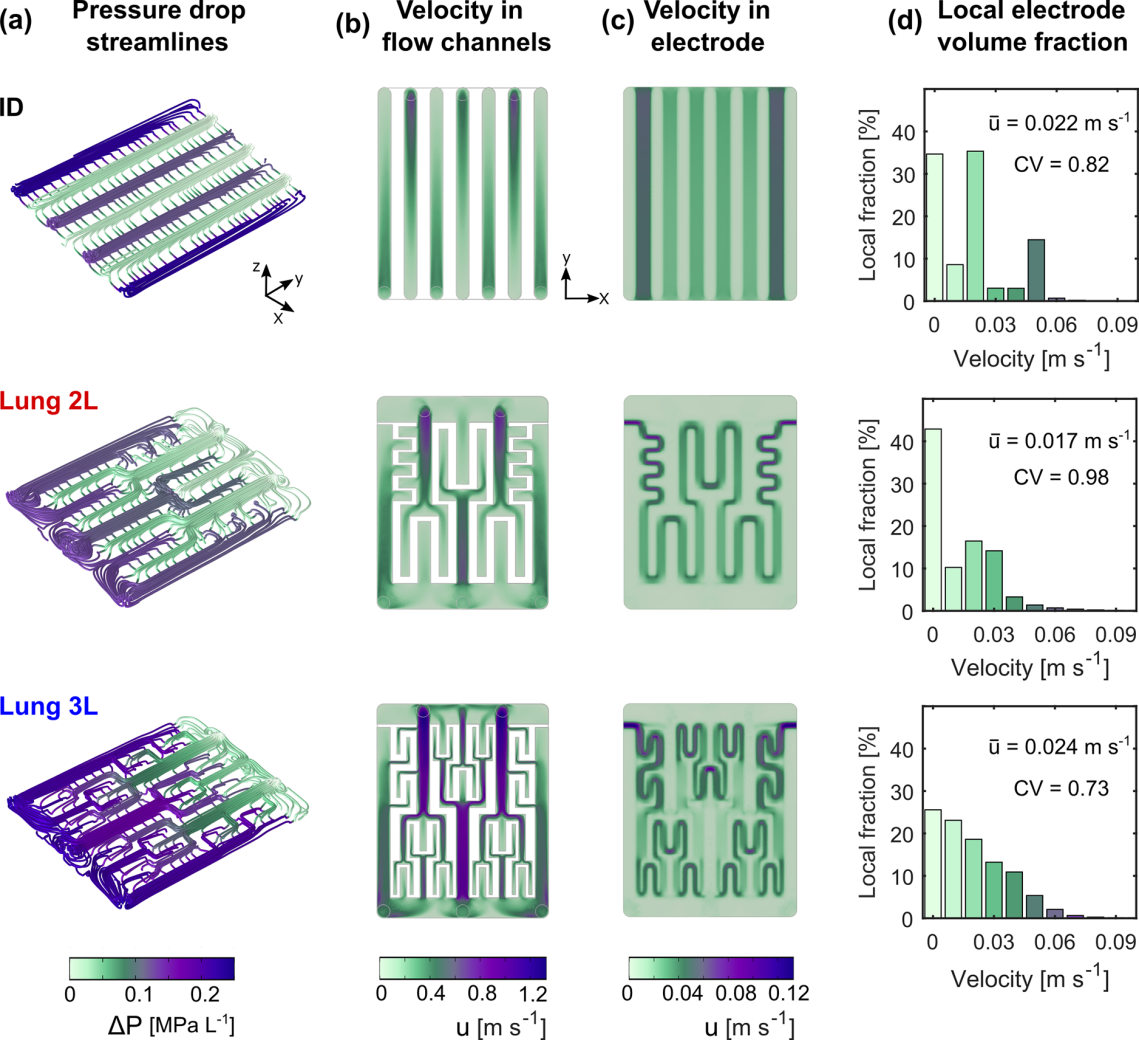
Fluid dynamics analysis of the ID, Lung 2L, and Lung 3L flow fields. (a) Streamlines together with pressure values as color expression limiting the representation of the streamlines between a minimum and maximum distance of 0.03 and 0.038 (fraction of the mean of the length of the bounding box of the geometry), respectively. Velocity color maps plotted at a planar cross section of (b) flow channels at half channel depth and (c) electrode at half the thickness. (d) Percentage of local electrode volume that sustains a certain electrolyte velocity, indicating the mean velocity *u̅* and the corresponding coefficient of variation CV, calculated as the ratio between the standard deviation and the mean velocity. In all the simulations, the inlet flow rate was set according to a theoretical electrolyte velocity in the electrode of 3.5 cm s^–1^.

The continuum 3D half-cell model is useful to provide a deeper understanding of the experimental measurements. In Figure 4, we present the fluid dynamic numerical results in the porous electrode and flow field domains for the three studied flow fields. The streamlines together with the relative pressure values (Figure 4a) help visualize the flow direction in the electrode. The results suggest that lung-inspired flow fields enhance the electrolyte distribution into the porous microstructure. A larger pressure drop is observed for the Lung 3L design compared to the Lung 2L design, which is in line with the experimental pressure drop measurements. As expected from overall mass conservation, the electrolyte velocity in the channels is approximately one order of magnitude higher than in the porous electrode for all flow fields (Figure 4b, c), resulting from the much smaller cross-sectional area of the channels compared to that of the electrode at the macroscopic level. The velocity contour plots in the porous electrodes show the non-uniform velocity field for the three flow fields ranging from 0 to 0.12 m s^–1^. Figure 4d reveals a wide range of electrolyte velocities encountered within the porous domain. The coefficient of variation of the averaged velocity in the electrode is quantified by the parameter CV (estimated as the ratio between the standard deviation and the mean velocity). Lung 2L gives the highest coefficient of variation (0.98), then ID (0.82), and last Lung 3L (0.73). These results demonstrate that assuming a constant velocity in the electrode when performing theoretical calculations implies a significant deviation from the real velocity field. Besides, the averaged velocity obtained from the simulations indicate that lower average values exist in the cell in comparison with the 3.5 cm s^–1^ estimated from eq 2. This must be considered not only for the lung-inspired geometries but also when using other types of interdigitated, serpentine, or flow-through flow fields.

### Electrochemical Performance

As shown in previous sections, before performing the electrochemical characterization of the different flow fields, the performance of the 3D printed ID was compared to the ID graphite flow field (Figure 2) to evaluate the performance in comparison with a state-of-the-art bipolar plate material. While the higher ohmic resistance of the printed flow fields is detrimental to the overall performance, it is a valid baseline material for this study which focuses on understanding mass transport in non-conventional flow field geometries. Although the proposed method is effective in manufacturing conductive structures, the use of costly coatings (such as Ag and Pt) is incompatible with large scale manufacturing. Our goal is to demonstrate the potential of these novel flow field geometries, and future work should be devoted to the development of coatings with lower electronic resistivity or readily conductive resins, which are cost effective.^39,61^ We acknowledge that the kinetic rate constant of the Fe^2+^/Fe^3+^ redox reaction on the surface of the platinum coating might be different compared with that on the carbon electrode surfaces.^62−65^ However, we do not anticipate the Pt coating to largely change the overall performance since the redox reaction is an outer sphere, and kinetic rate constants in the literature do not differ significantly. Furthermore, the area of the carbonaceous porous electrode is significantly larger than the area of the flow field coated with platinum. Additionally, to provide a robust comparison, the newly printed flow fields are compared against a baseline ID flow field which also has been fabricated following the same manufacturing process, including coating with Pt. In addition, the electrochemical experiments performed in this work using single electrolyte flow cells were limited within a stable electrochemical window to avoid any gas evolution,^66^ and these were not observed during the experiments.

**Figure 5:**
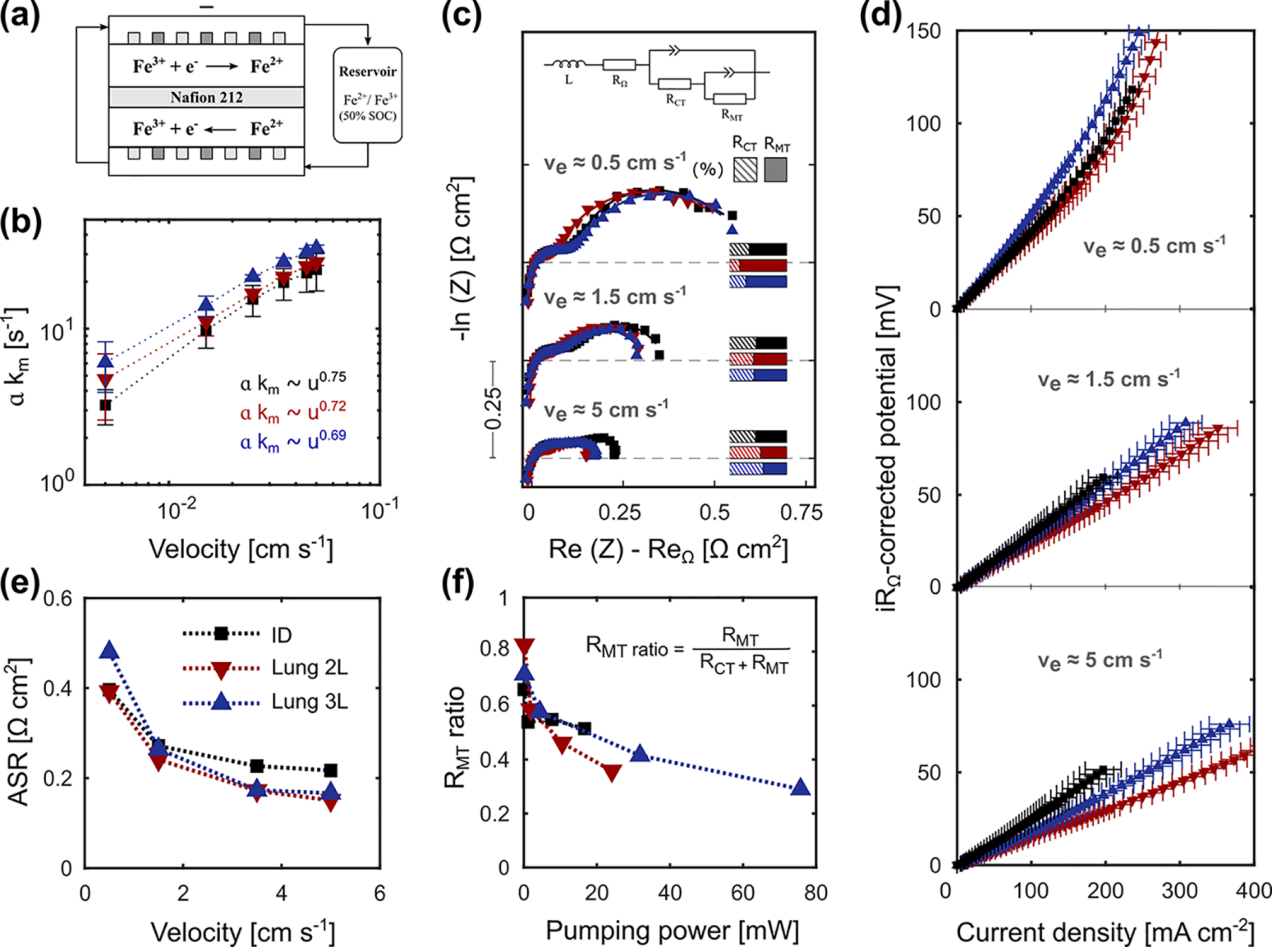
Electrochemical characterization of the three 3D printed flow fields. (a) Schematic of the single-electrolyte flow cell used for the experimental characterization. (b) Volume-specific surface area mass transfer coefficients obtained from limiting current measurements over a range of velocities. (c) Nyquist plots together with bars accounting for the percentage of charge and mass transport resistances. (d) *iR*_Ω_-corrected polarization at 0.5, 1.5, and 5 cm s^–1^ electrolyte velocities. (e) Area-specific resistance at different electrolyte velocities. (f) Mass transport resistance against the required pumping power for every electrolyte velocity. All resistances in the Nyquist plot, including the ASR and RMT ratio, are normalized by the geometrical area of the electrode (2.55 cm^2^).

**Figure 6:**
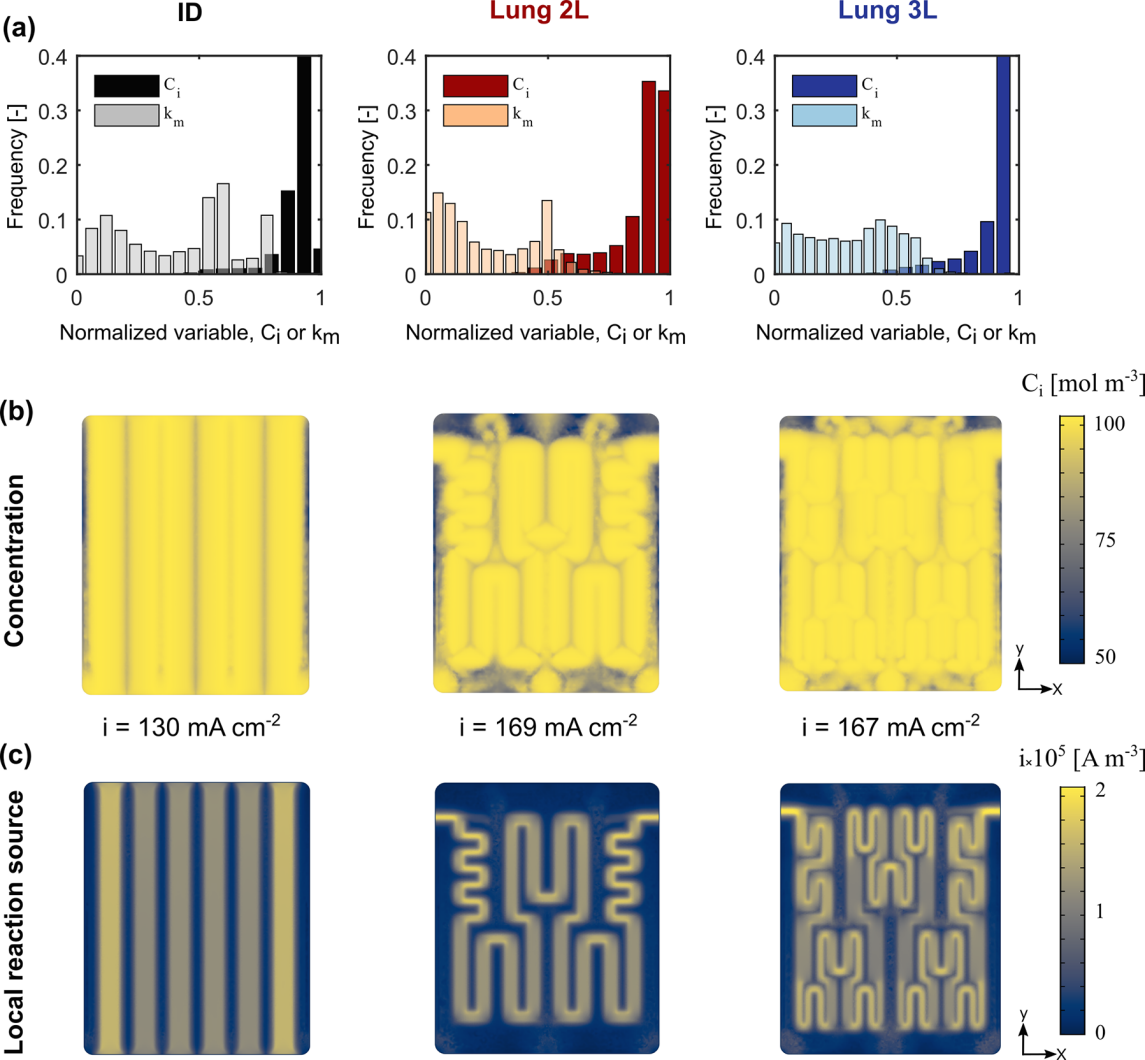
Electrochemical analysis of the ID (left), Lung 2L (middle), and Lung 3L (right) flow fields. (a) Normalized distributions of local *k*_m_ and *C*_Fe^2+^_ (by the maximum local value), in the 3D electrode domain using a 10 × 200 × 200 grid (thickness × length × width points). (b) Concentration contours at 0.2 V and 3.5 cm s^–1^ electrolyte velocity together on contours with the current density values extracted from the current collectors in a cut section at half of electrode thickness. Inlet concentration ratio of 0.1/0.1 M ratio for *C*_Fe^2+^,0_/*C*_Fe^3+^,0_. (c) Local reaction source at the limiting conditions of 0.4 V, 1.5 cm s^–1^ electrolyte velocity and 0.6/3 mM ratio for inlet concentrations *C*_Fe^2+^,0_/*C*_Fe^3+^,0_ in a cut section at half of electrode thickness.

The single-electrolyte flow cell setup was used to evaluate the electrochemical performance under realistic operating conditions (Figure 5a). To characterize the mass transport losses, limiting current experiments were carried out to obtain the volume-specific surface area mass transfer coefficient (*a k*_m_) of every flow field-carbon paper system. This experiment leverages the reduced active species concentration such that after a certain applied potential, the concentration at the electrode surface can be assumed zero, leading to a plateau on the polarization curve^67^ (Figure S12). The value of *a k*_m_ can be obtained by conducting a control volume analysis and assuming a constant bulk concentration according to ref (68).
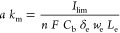
13where *I*_lim_ is the measured limiting current, *C*_b_ is the active species concentration in the bulk electrolyte, δ_e_ is the electrode thickness, and *w*_e_ and *L*_e_ are the electrode width and length, respectively. Figure 5b shows the product of the electrode specific surface area and the mass transfer coefficient (*a k*_m_) for the ID, Lung 2L, and Lung 3L, over a range of electrolyte velocities. Lung 3L features the highest mass transfer coefficients among the three flow fields. However, when comparing against the required pressure drop (Figure S13), similar values and trends are observed for the three flow fields, which is attributed to the higher flow rates required by lung-inspired flow fields. To gain insight into these differences, we use the 3D continuum model to simulate the limiting-reactant case (0.3/6 mM for the inlet concentrations of Fe^2+^/Fe^3+^, respectively). In Figure 6a, a frequency analysis of *a k*_m_ and concentration values of Fe^2+^ is performed, taking 400,000 local points in the 3D electrode volume for a reliable examination. The *a k*_m_ and *C*_Fe^2+^_ were normalized by their maximum value in the 3D domain. In light of the numerical results, we ascribe the high mass transfer rate of Lung 3L to a more homogeneous electrolyte distribution, as supported by the more uniform values of *a k*_m_ in Figure 6a over the entire 3D domain. These results align with findings from other works, where fractal interdigitated geometries were found to provide excellent electrolyte distribution.^29^ On the contrary, the ID and the Lung 2L feature greater dispersion of *a k*_m_ values as noticed in the frequency plots. This can be related to differences in the electrolyte-exchange perimeter (Lung 3L has 183.9 mm while ID and Lung 2L have 96.0 and 113.1 mm, respectively). This directly impacts the homogeneity of the species mass transport (Figure 6b), leading to reactant starvation in certain electrode regions, which experimentally will result in a lower *a k*_m_ for the tandem electrode-flow field system. Contour plots of concentration (Figure 6b) and local current source (Figure 6c) demonstrate the greater flow uniformity that Lung 3L induces in the porous electrode. Contour plots of the local current density are equivalent to those of *k*_m_ as, at the evaluated conditions (0.6/3 mM of Fe^2+^/Fe^3+^, 1.5 cm s^–1^, and 0.4 V), mass transfer is dominating the system performance.

To deconvolute resistive contributions to cell polarization, Nyquist plots (Figure 5c) are used to represent the electrochemical impedance spectroscopy results (ohmic, kinetic, and mass transport overpotential losses). The ohmic resistance of each cell was obtained from the high-frequency intercept which accounts for the contact and membrane resistances in the system and is subtracted from the total resistive losses (see Section S10). Since the charge and mass transfer resistances are complex to deconvolute, an equivalent circuit model^69−71^ was used to fit the experimental values (Tables S8–S11). As shown on the top part of Figure 5c, the model consists of an inductor (*L*) in series with an ohmic resistor (*R*_Ω_), as well as a constant-phase element (CPE) in parallel with a charge transfer resistor (*R*_CT_), in series with a second CPE in parallel with a mass transfer resistance (*R*_MT_). As expected, increasing the electrolyte velocity significantly enhances the mass transport of species,^72−74^ resulting in a reduced mass transfer resistance from 0.5 Ω cm^2^ (at *v*_e_ ≈ 0.5 cm s^–1^) to 0.25 Ω cm^2^ (at *v*_e_ ≈ 5 cm s^–1^) for the ID flow field. The mass transfer resistance of both Lung 2L and 3 L designs are lower than that of the ID design. In general, the charge transfer resistance (*R*_CT_) of Lung 2L is lower than that of ID and Lung 3L and is less affected by the electrolyte velocity. We hypothesize that Lung 2L is accessing a larger electrode reaction volume due to the lower charge transfer resistance observed from Figure 5c (the resistances deconvolution is shown in Tables S8–S11).

To further analyze the kinetic and mass-transfer overpotential losses, the cell polarization was plotted, corrected for the ohmic contribution. Figure 5d shows the current density output at an applied *iR*_Ω_-corrected potential for different imposed electrolyte velocities in the electrode. At all velocity conditions, Lung 2L exhibits the highest electrochemical performance, which becomes remarkable at high velocities, such as *v*_e_ ≈ 5 cm s^–1^ where the Lung 2L provides ∼350 mA cm^–2^ while the traditional ID gives ∼200 mA cm^–2^ at 50 mV of *iR*_Ω_-corrected potential. From the distribution of normalized concentration values (*C*_Fe^2+^_) obtained from the simulations (Figure 6a), it is observed that a larger amount of Fe^2+^ is being consumed in the electrode when using Lung 2L flow field (i.e., more flattened distribution profiles which reaches lower values of normalized *C_i_*). These results suggest that the structural pattern of lung-inspired designs improves the electrode volume utilization compared to the ID flow field. Besides, the wider ribs and lower channel contact area enhances the electrolyte under-rib convection,^75,76^ accessing larger electrode volumes for the Lung 2L than the Lung 3L. Lung 3L also demonstrates a better performance than the traditional ID, reaching a current of ∼250 mA cm^–2^ at 5 cm s^–1^. Interestingly, at the lowest electrolyte velocity (*v*_e_ ≈ 0.5 cm s^–1^), the Lung 3L performs the worst. We hypothesize that this is because the required flow rate is not sufficient to completely fill the flow channels, resulting in unutilized electrode reaction volume.

For deepening our understanding, charge and mass transport overpotential losses are deconvoluted and analyzed by calculating the area-specific resistance (ASR)^77,78^ and mass transport resistance (*R*_MT_), respectively. ASR values were obtained from the slope of the *iR*_Ω_-corrected polarization curves and can give information about the accessible ECSA, activation and mass transport overpotentials and possible ohmic losses coming from the distributed resistance caused by the ohmic resistance in the cell.^56,57^ Despite the slightly higher values of ASR were found for the ID flow field, Figure 5e shows similar trends for the minimization of ASR over the increase of the electrolyte velocity for the three flow fields. This reveals a greater electrolyte penetration in the electrode improving the reactant supply, leading to a subsequent decrease on mass transport resistance and ASR.^45^ On the contrary, Figure 5f reveals greater sensitivity of the mass transport resistance in lung-inspired designs with respect to flow velocity than in traditional ID flow fields, which results in lower *R*_MT_ values, proving the better distribution of species throughout the electrode. Furthermore, the pumping power required by the Lung 2L to minimize *R*_MT_ values is almost three times lower than that required by the Lung 3L.

### Performance Trade-Offs

**Figure 7:**
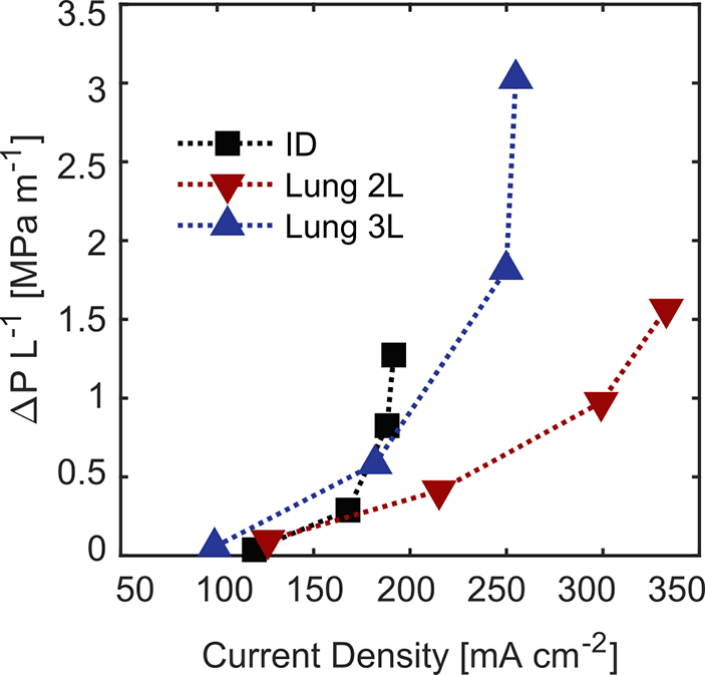
Normalized pressure drop versus current density at 50 mV *iR*_Ω_-corrected potential for the three different flow fields. The markers correspond to 0.5, 1.5, 3.5, and 5 cm s^–1^ electrolyte velocities.

Identifying reactor operational windows for various electrode-flow field combinations is necessary to maximize energy efficiency. In Figure 7, we represent the competing requirements of maximizing the electrochemical performance and minimizing the pressure losses through each flow field-electrode configuration. We compute the pressure drop per unit length required to sustain a certain electrolyte velocity and plot this metric versus the corresponding current density obtained from the flow cell at 50 mV *iR*_Ω_-corrected potential. The reader should note that the potential values represent overpotentials (i.e., losses), thus the higher the current density at a given potential would result in overall higher electrochemical performance. Each data point represents different electrolyte velocities (0.5, 1.5, 3.5, and 5 cm s^–1^). Among the studied flow field geometries, the Lung 2L in combination with carbon paper electrode provides the best trade-off for all electrolyte velocities. It achieves current densities of ∼340 mA cm^–2^ at the pressure drop of 1.5 MPa m^–1^, which is approximately half of the pressure drop required for Lung 3L. Even though Lung 3L provides greater current densities than ID, the associated pressure loss is significantly higher, making the balance unfavorable. While increasing the electrolyte exchange perimeter, as in the case of Lung 3L, improves the homogeneous distribution of the electrolyte providing higher mass transfer rates, the pressure loss associated has a detrimental impact on the system efficiency. As a consequence, a balance must be found between high exchange perimeter for optimum reactant distribution and wide ribs for large accessibility of electrode reaction volume.

## Conclusions

Emerging electrochemical systems, such as redox flow batteries, require the development of tailored flow field geometries to sustain the stringent requirements of the electrochemical cell. While existing flow field geometries have been repurposed from fuel cells and are functional, the bottom-up design and manufacture of tailored flow field architectures is required to further advance the technology. In this work, we demonstrate that lung-inspired designs, which are based on interdigitated patterns with fractal geometrical characteristics and a higher electrolyte-exchange perimeter, outperform traditional flow fields and hold a promise for advanced flow battery designs. We propose a two-step manufacturing process consisting of stereolithography 3D printing and conductive coatings. Using this method, we manufacture two lung-inspired flow field geometries with increasing levels of branching. By employing a series of pressure drop measurements, electrochemical diagnostics, and 3D numerical simulations of one half-cell, we demonstrate the suitability of the proposed lung-inspired flow field geometries. In general, lung-inspired designs enable a better reactant distribution throughout the electrode, resulting in higher mass transfer rates. Moreover, the pressure drop in these designs remains in the same order of magnitude as for interdigitated flow fields even though the electrolyte-exchange perimeter is larger. Particularly, we find that the Lung 2L provides the best electrical and pumping power trade-off due to its high accessibility to the electrode reaction volume and low hydraulic resistance. Although the Lung 3L distributes the electrolyte more uniformly, the higher required pumping power challenges the performance trade-off. Overall, we conclude that lung-inspired flow fields are a promising alternative flow field pattern to combine with carbon paper electrodes. Future work should be devoted to topology optimization in combination with experiments to find the optimal channel and rib width as well as the number of branches to optimize lung-inspired flow fields.

## Supporting Information Available

CAD drawings; determination of the electrolyte exchange perimeter; flow cell configuration; reproducibility study of electrochemical experiments; determination of the electrochemically active surface area; details of the numerical model in COMSOL Multiphysics and experimental validation; electrical conductivity measurements; apparent permeability and Forchheimer equation fits; limiting current measurement example; volume-specific surface area mass transfer coefficients vs pressure drop; and electrochemical impedance spectroscopy fittings (PDF)
