# Supplementary material for: Engineering Lung-Inspired Flow Field Geometries for Electrochemical Flow Cells with Stereolithography 3D Printing
Source: ACS Sustain Chem Eng. 2023 Jul 24;11(33):12243–55. doi: 10.1021/acssuschemeng.3c00848 (PMC10445267; doi:10.1021/acssuschemeng.3c00848)
Supplement: Supplementary file 1 — sc3c00848_si_001.pdf [file sc3c00848_si_001.pdf]

# Supporting Information

# Engineering Lung-inspired Flow Field Geometries for Electrochemical Flow Cells with Stereolithography 3D Printing

Vanesa Muñoz-Perales †, Maxime van der Heijden ‡, Pablo A. García-Salaberri †, Marcos Vera † and Antoni Forner-Cuenca *,‡

† *Department of Thermal and Fluids Engineering, Universidad Carlos III de Madrid, 28911-Leganés, Spain*

‡ *Electrochemical Materials and Systems, Department of Chemical Engineering and Chemistry, Eindhoven University of Technology, Eindhoven 5600 MB, The Netherlands*

* *Corresponding author:* a.forner.cuenca@tue.nl

*Number of pages: 17*

*Number of figures: 14*

*Number of tables: 11*

Section S1 – CAD drawings and determination of the electrolyte exchange perimeter (PE) ....... 2

Section S2 – Materials and chemicals ....... 4

Section S3 – Reproducibility study of electrochemical experiments ....... 5

Section S4 – Numerical model in COMSOL Multiphysics ....... 6

S4a - Geometry and mesh of the half-cell model ....... 6

S4b - Species concentration at the electrode surface ....... 6

S4c - Estimation of the electrolyte potential at the electrode-membrane interface ....... 7

S4d – Mesh independence analysis ....... 8

S4e – Validation of the numerical model ....... 9

Section S5 – Determination of the electrochemically active surface area (ECSA) ....... 10

Section S6– Conductivity calculation of graphite and printed flow fields ....... 12

Section S7 – Hydraulic analysis: apparent permeability and Forchheimer equation fits ....... 13

Section S8 – Limiting current measurements ....... 14

Section S9 – Volume-specific surface area mass transfer coefficient vs. pressure drop ....... 14

Section S10 – Electrochemical impedance spectroscopy fittings ....... 15

## Section S1 – CAD drawings and determination of the electrolyte exchange perimeter ($P_E$)

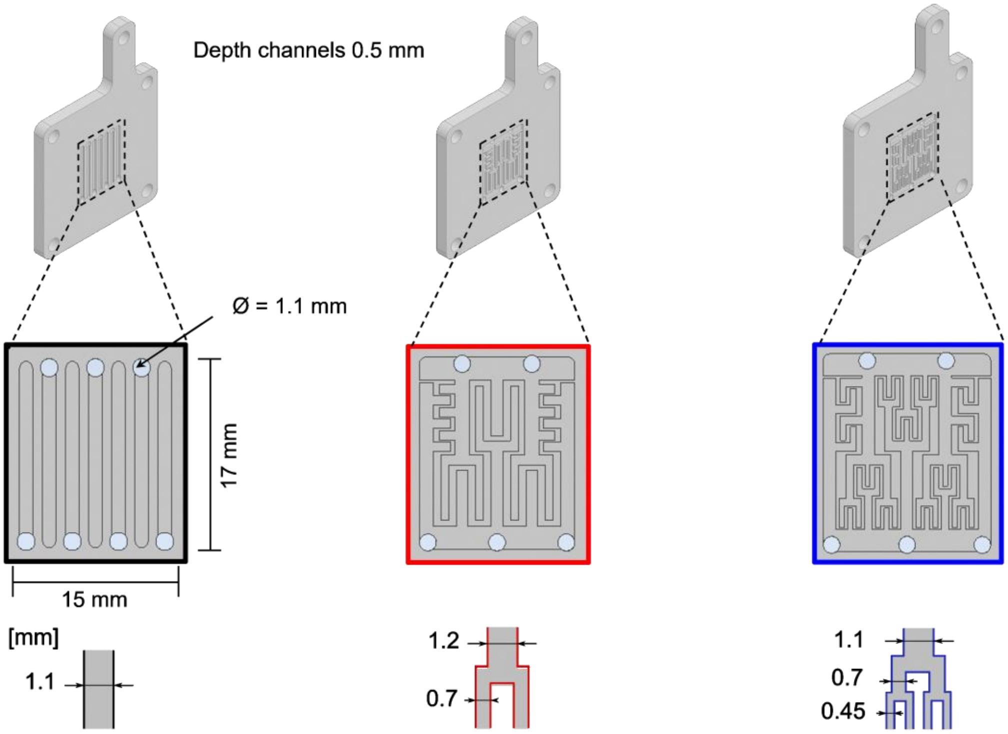


**Figure S1:** CAD drawings of the printed flow fields together with the dimensions of the channel levels, channels depth and inlet/outlet holes.

To calculate the electrolyte exchange perimeter, an average is taken between the inner (Figure S2a) and outer (Figure S2b) perimeter, excluding the outer edges close to the cell border.

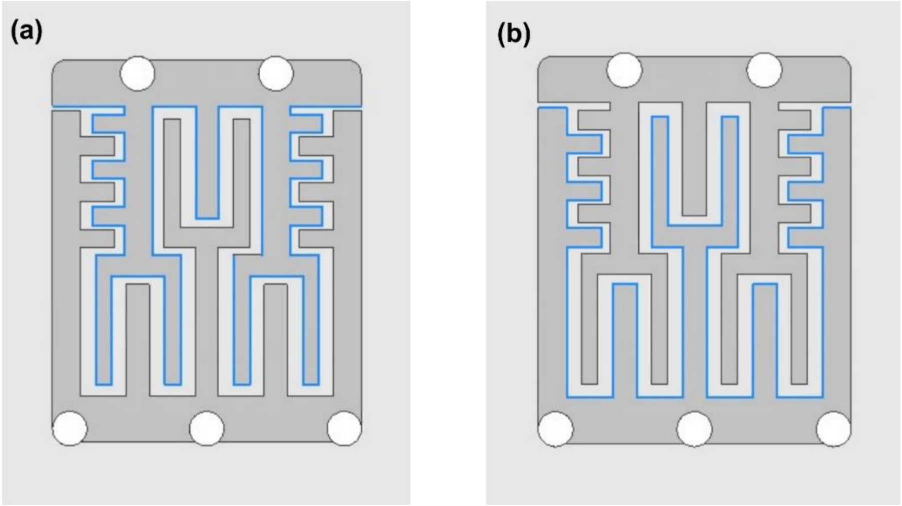


**Figure S2:** Electrolyte exchange perimeter definition: (a) inner perimeter and (b) outer perimeter.

**Table S1.** Inner and outlet perimeter dimensions for the calculation of the averaged exchange perimeter.

| Symbol | Inner perimeter [mm] | Outer perimeter [mm] |
|---|---|---|
| ID | 96 | 96 |
| Lung 2L | 110.9 | 115.3 |
| Lung 3L | 172.9 | 194.9 |

## Section S2 – Materials and chemicals

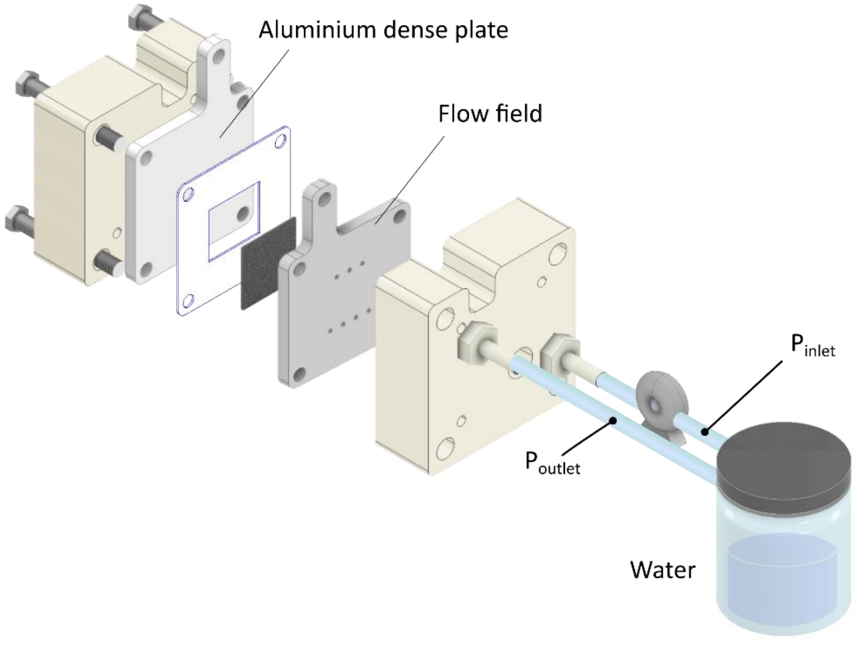


**Figure S3:** Cell configuration for the pressure drop experiments.

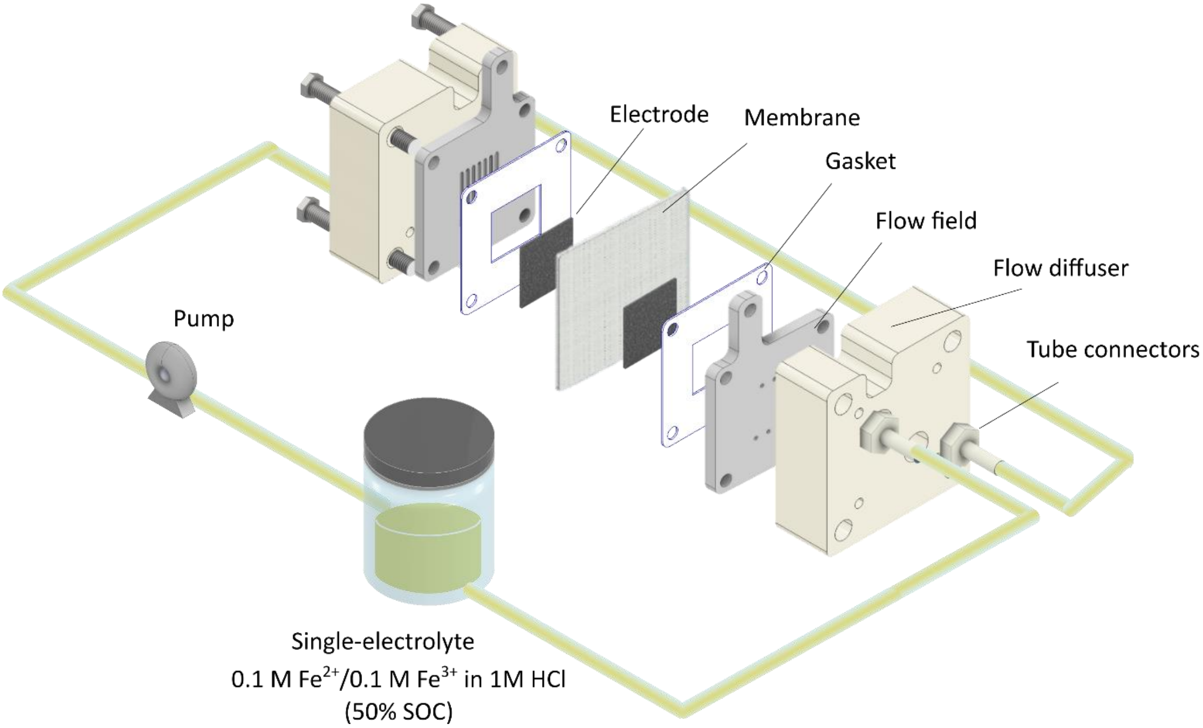


**Figure S4:** Single-electrolyte cell configuration for the electrochemical experiments and cell components.

## Section S3 – Reproducibility study of electrochemical experiments

To estimate the reproducibility of the polarization and impedance experiments, seven different cells were tested with the ID flow field in combination with the carbon paper electrode. The fresh materials (electrodes, membrane and electrolytes) were replaced between experiments.

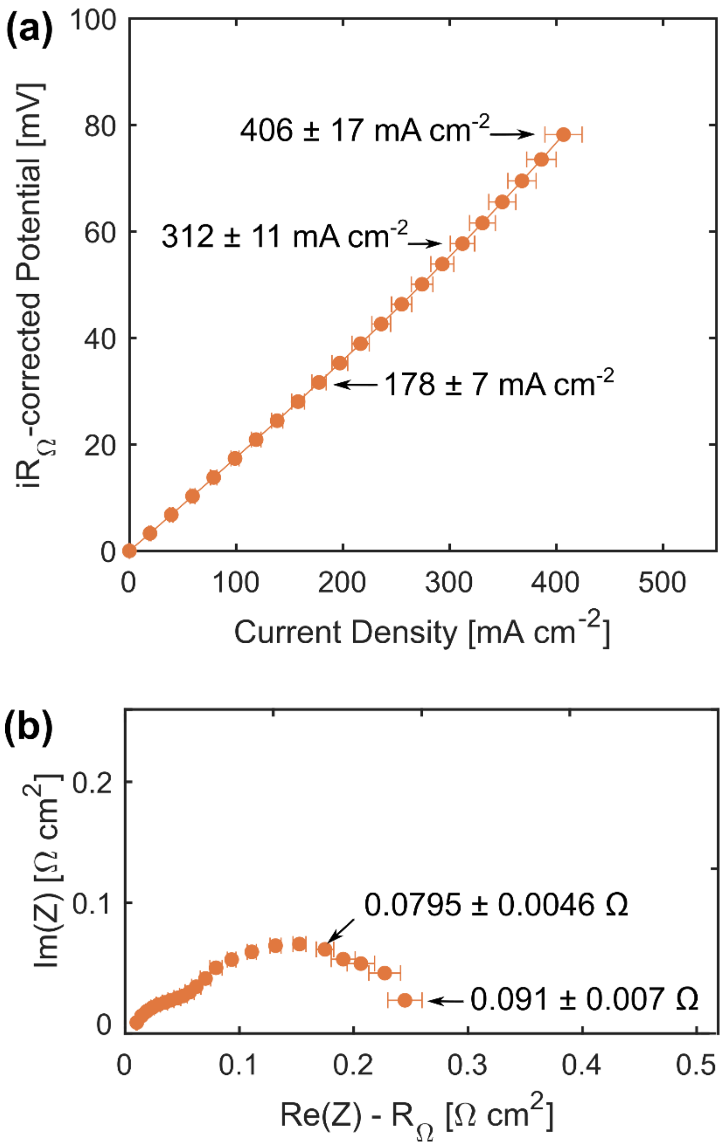


**Figure S5.** Reproducibility of experiments evaluated with the combination of ID flow field and carbon paper electrode at 1.5 cm $s^{-1}$ repeating the experiment in seven different cells with the same configuration. (a) $iR_{\Omega}$-corrected potential vs. current density and (b) Nyquist plot.

## Section S4 – Numerical model in COMSOL Multiphysics

### S4a - Geometry and mesh of the half-cell model

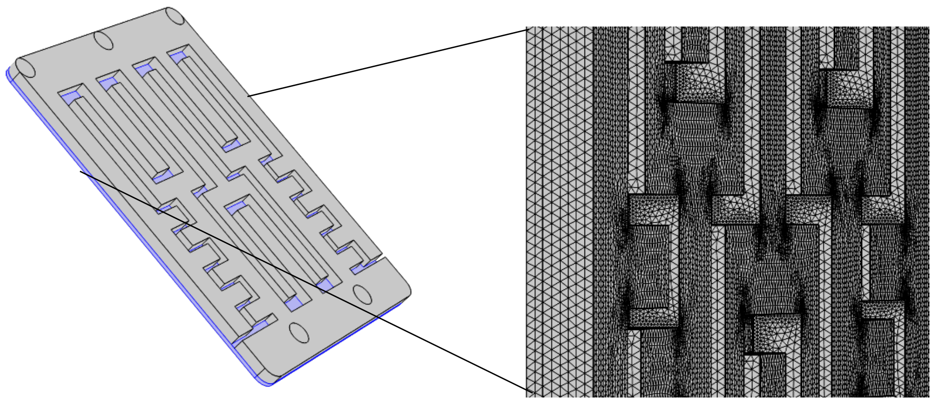

**Figure S6:** Geometry and mesh of the modeling domain in COMSOL 5.6 Multiphysics® for the Lung 2L flow field. The electrode domain is represented with the purple color and the flow field domain with the gray color. The mesh consists of 3401210 elements.

### S4b - Species concentration at the electrode surface

To calculate the concentration of species at the surface of the electrode, the mass transfer flux of $Fe^{2+}$ and $Fe^{3+}$ species from the electrolyte bulk to the electrode surface is modeled by assuming a linear Nernst diffusion layer:

$$N_j^S = k_m(C_j - C_j^s) = \frac{i_{loc}}{F} \tag{S1}$$

where $j$ refers exclusively to the redox species $Fe^{2+}$ and $Fe^{3+}$.

Substituting $i_{loc}$ using Eq. (10) in the main article, yields a system of two linear equations whose solution gives the species concentrations as:

$$C_O^S = \frac{\left(1 + \frac{A}{k_m}\right) C_O + \frac{A}{k_m} C_R}{1 + \frac{A}{k_m} + \frac{B}{k_m}} \tag{S2}$$

$$C_R^S = \frac{\left(1 + \frac{B}{k_m}\right) C_R + \frac{B}{k_m} C_O}{1 + \frac{A}{k_m} + \frac{B}{k_m}} \tag{S3}$$

where the value of $k_m$ is assumed the same for the oxidized (O) and reduced (R) redox species, and the coefficients A and B are given by:

$$A = k^0 C_R^{\alpha_c - 1} C_O^{\alpha_a} \, exp\left(\frac{\alpha_a F}{RT}\eta\right) \quad (S4)$$

$$B = k^0 C_R^{\alpha_c} C_O^{\alpha_a - 1} \; exp\left(\frac{-\alpha_c F}{RT}\eta\right) \quad (S5)$$

## S4c - Estimation of the electrolyte potential at the electrode-membrane interface

The electrolyte potential at the membrane interface can be estimated considering a voltage loss across the membrane of $\Delta\Phi_m$ [1]:

$$\Delta\Phi_m = R_m I_m \quad (S6)$$

Where $I_m$ is the ionic current passing through the membrane and $R_m$ the membrane resistance, which can be calculated from the membrane conductivity as:

$$R_m = \frac{\delta_m}{\sigma_m A_m} \quad (S7)$$

where $\delta_m$ and $A_m$ are the membrane thickness and cross-sectional area, respectively, and $\sigma_m$ is the membrane conductivity. This results in a membrane resistance value of 0.0356 Ω calculated with the data from **Table S2.**

**Table S2.** Membrane parameters.

| **Symbol** | **Quantity** | **Value** | **Origin** |
|---|---|---|---|
| $\delta_m$ | Thickness [m] | $5.08\times10^{-5}$ | Datasheet [2] |
| $A_m$ | Cross-section area [$m^2$] | $2.55\times10^{-4}$ | Measured |
| $\sigma_m$ | Conductivity [$S\ m^{-1}$] | 5.6 | [3] |

The liquid potential at the membrane interface is calculated following the next steps:

1. An arbitrary value of $V_{cell}$ = 0.3 V is chosen for the estimation of $\Phi_L$ for the ID case
2. Give a random value to $\Phi_L$ at membrane. E.g. 0 V for Vcell = 0.3 V
3. Simulation → obtain $I_m$ = 573.6 A $m^{-2}$ * 2.55 $\times10^{-4}$ $m^{-2}$ = 0.146 A
4. Calculation of $\Phi_L(\text{Eq. S6}) = \Delta\Phi_m$ = 0.146 * 0.0356 = $5.41\times10^{-3}$ V = 5.52 mV

## S4d – Mesh independence analysis

The mesh independence analysis was performed at $V_{cell}$ = 0.1 V and 3.5 cm $s^{-1}$ electrolyte velocity. As observed from **Table S3** and **Figure S7** an stable solution is achieved after 4 000 000 number of elements, which is reflected in the monitorization of the current density and on the 2D concentration profiles of $Fe^{3+}$.

**Table S3.** Mesh evaluation with different number of elements and the resulting current density output from the cell and concentration profiles of $Fe^{3+}$.

| **Elements** | **104314** | **4260497** | **8921675** |
|---|---|---|---|
| Mesh drawing | 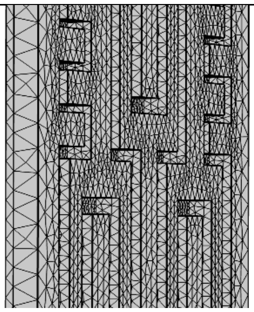 | 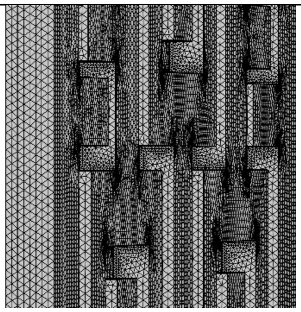 | 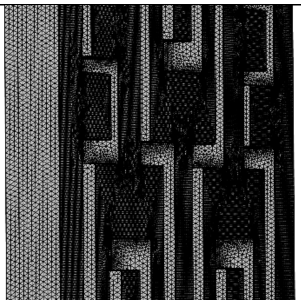 |
| Current density | 71.3 | 81.3 | 81.5 |
| 2D plots concentration $Fe^{3+}$ | 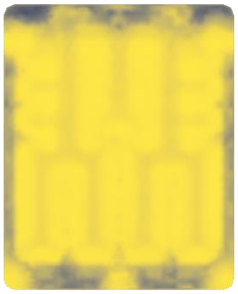 | 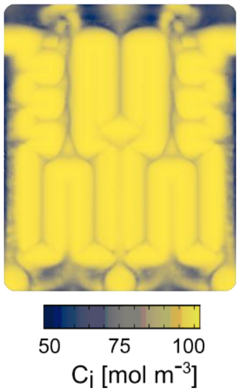  | 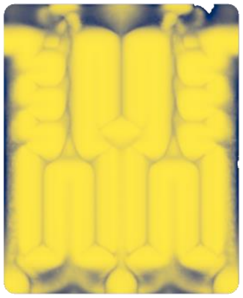 |

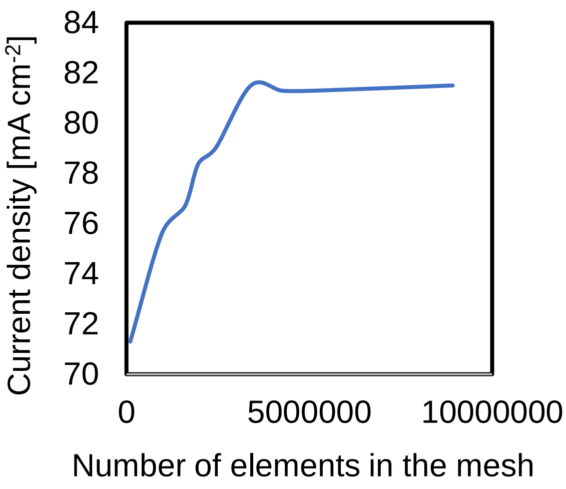


**Figure S7:** Mesh independence analysis: the response in the total current density from the electrochemical flow cell is evaluated with different number of elements in the mesh.

## S4e – Validation of the numerical model

The model was successfully validated for the three flow field designs at an electrolyte velocity of 3.5 cm $s^{-1}$. The cell polarization curves were evaluated experimentally and compare to the model predictions. Since the model formulation does not include the ohmic losses from the flow fields contact resistance, we corrected the cell polarization predictions using the measured ohmic resistance, i.e., 0.496 Ω $cm^2$ for the printed flow field (**Figure 2b**) in the empty cell where the two flow fields are stacked together (**Figure S11b**).

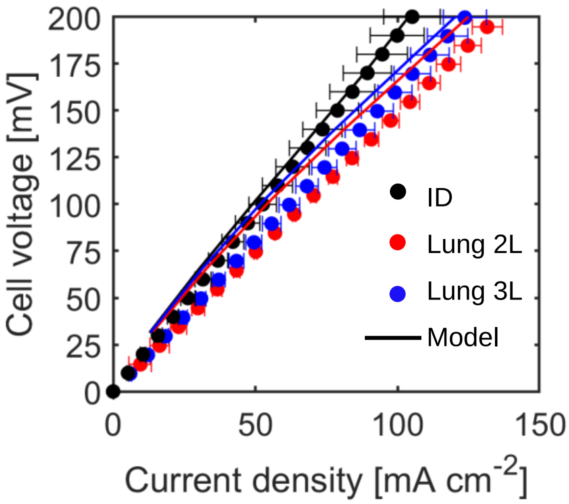


**Figure S8:** Validation of the numerical model for the three different flow fields at the electrolyte velocity of 3.5 cm $s^{-1}$.

## Section S5 – Determination of the electrochemically active surface area (ECSA)

The electrochemically active surface area of the electrode was estimated by charging the double layer in a flow cell setup as illustrated in **Figure S4**, using the graphite interdigitated flow field. To do so, a supporting electrolyte of 2 M HCl in water was used to avoid the occurrence of faradaic processes. A linear electrolyte velocity of 5 cm $s^{-1}$ was used and cyclic voltammetry was performed between -0.2 V and 0.2 V at 5 different scan rates (20, 50, 100, 150, and 200 mV $s^{-1}$). The specific capacitance of glassy carbon (18 µF $cm^{-2}$ [4]) was used to estimate the ECSA based on prior literature.[5,6] A typical voltammetry result, representative for the rest of the data in the study, is shown in **Figure S9**.

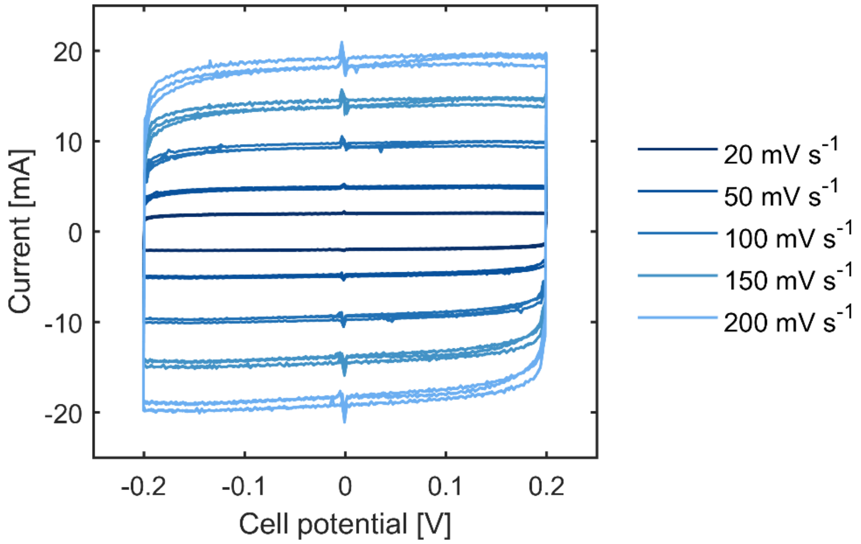


**Figure S9.** Cyclic voltammetry measurements at multiple scan rates (20, 50, 100, 150 and 200 mV $s^{-1}$) of a flow cell containing regular interdigitated flow fields with carbon paper electrodes. The electrolyte solution was 2 M HCl in water. Three repetitions of the measurement were performed with three different cells (n = 3).

From the cyclic voltammetry measurements, the average capacitive current can be extracted at 0 V for each of the scan rates. Subsequently, the electrochemical double layer capacitance (EDLC) can be extracted from the slope of the corresponding linear fit, according to the following equation [7]:

$$I = EDLC\frac{dV}{dt} \quad (S8)$$

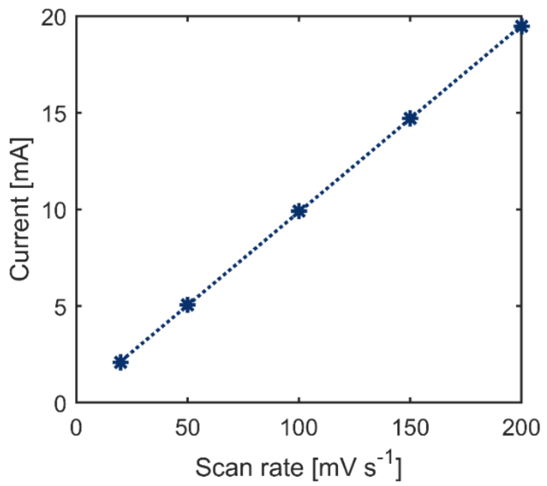


**Figure S10.** Linear fitting of the average capacitive current at different scan rates to obtain the EDLC from the slope.

## Section S6– Conductivity calculation of graphite and printed flow fields

Two different cell configurations were used as explained in **Figure S11** to estimate the total ohmic resistance in the regular cell with all components (**Figure S11a**) and exclusively of the flow fields to isolate the ohmic resistance coming from the flow fields due to the different graphite or printed materials (**Figure S11b**).

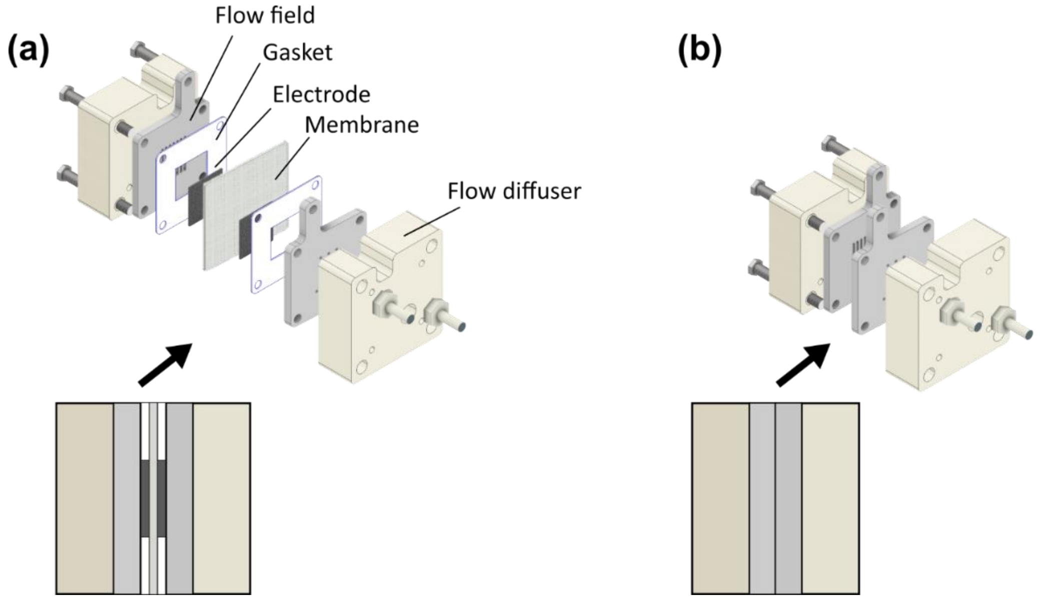


**Figure S11:** Cell setup used for the ohmic resistance measurement and subsequent conductivity estimation. (a) Regular cell with all components including electrolyte, electrodes, gaskets and membrane. (b) Empty cell without electrolyte, electrodes, gaskets and membrane.

The electrical conductivity of the flow fields (**Table S4**) was calculated from the ohmic resistance measured in the empty flow cell setup with only the flow fields (**Figure S11b**). Electrochemical impedance spectroscopy measurements were performed following the same procedure as explained in the methodology section of the manuscript, and the high-frequency intercept was taken as the ohmic resistance value for the conductivity estimation according to

$$\sigma_{FF} = \frac{L}{A\,R} \tag{S9}$$

where $\sigma_{FF}$ is the electrical conductivity [S $m^{-1}$], A the contact area (taken as that of the electrode 2.55 $cm^2$), L the thickness the sample (2 x flow field thickness (3.18 mm)) and R the measured resistance from EIS [Ω].

**Table S4:** Conductivity values of the graphite and printed flow fields estimated from an empty cell with only the flow fields stacked together.

| **Empty cell only flow fields** | **Conductivity [S $m^{-1}$]** |
|---|---|
| Graphite flow field | 318 |
| Printed flow field | 128 |

## Section S7 – Hydraulic analysis: apparent permeability and Forchheimer equation fits

**Table S5:** Hydraulic parameters of the different flow fields with Freudenberg carbon paper obtained from Darcy-Forchheimer fittings.

| **Flow Field** | **$k \cdot 10^{-11}$ [$m^2$]** | **$\beta \cdot 10^5$ [$m^{-1}$]** |
|---|---|---|
| ID | 7.23 ± 1.88 | 1.76 ± 0.03 |
| Lung 2L | 8.07 ± 1.81 | 2.85 ± 0.02 |
| Lung 3L | 3.80 ± 0.22 | 6.01 ± 0.12 |

**Table S6:** Percentage of the flow field and electrode contribution to the total pressure drop.

| **Flow Field** | **% FF / E** |
|---|---|
| ID | 53/47 |
| Lung 2L | 59/41 |
| Lung 3L | 73/27 |

## Section S8 – Limiting current measurements

To capture the effect of flow field geometry on the mass transfer polarization region, limiting current measurements were performed to estimate later the volume-specific surface area mass transfer coefficients. **Figure S12** shows an example of limiting current measurements which are achieved after 0.3 V of applied cell potential.

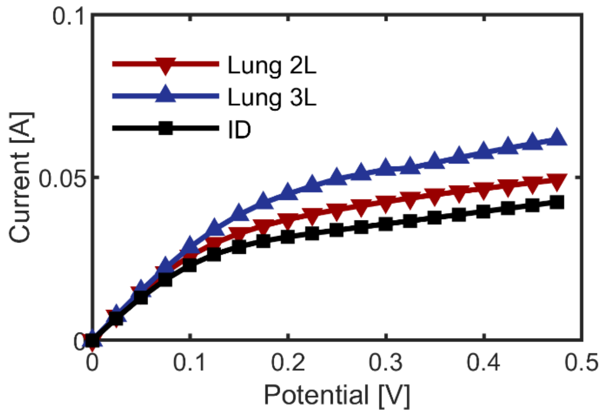


**Figure S12:** Limiting current measurements at an electrolyte velocity of 1.5 cm $s^{-1}$.

## Section S9 – Volume-specific surface area mass transfer coefficient vs. pressure drop

In **Figure S13** a comparison between the volume-specific surface area mass transfer coefficients against the corresponding pressure losses is presented for the three flow fields. No significant differences were observed due to the higher flow rates required by lung-inspired flow fields driven by their high electrolyte exchange perimeter.

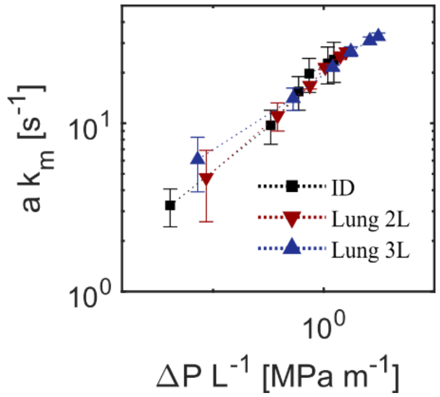


**Figure S13:** Volume-specific surface area mass transfer coefficients obtained from limiting current measurements over a range of pressure drop normalized by the electrode length (1.7 cm) corresponding to the different studied electrolyte velocities.

## Section S10 – Electrochemical impedance spectroscopy fittings

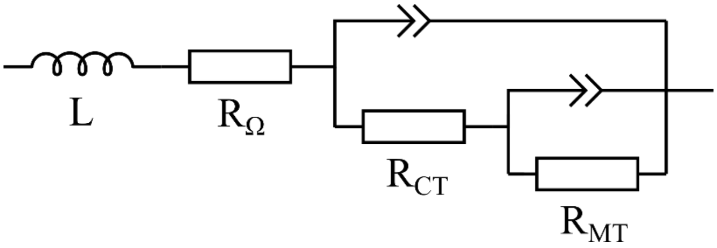


**Figure S14.** Equivalent circuit model for electrochemical impedance spectroscopy.

The Z fittings are made based on the average PEIS data of the experiments (2-3 repetitions) using E Z-fit tool in the software EC-lab V11.33.

**Table S7** – Fitted parameters from electrochemical impedance spectroscopy for the graphite-based and 3D printed interdigitated flow fields with the Freudenberg carbon paper electrode at the electrolyte velocity of 3.5 cm $s^{-1}$.

| Flow field | $R_{TOT}$ [Ω $cm^2$] | $R_{ohm}$ [Ω $cm^2$] | $R_{CT}$ [Ω $cm^2$] | $R_{MT}$ [Ω $cm^2$] |
|---|---|---|---|---|
| ID printed | 1.997 | 1.680 | 0.196 | 0.120 |
| ID graphite | 0.485 | 0.329 | 0.082 | 0.074 |

**Table S8** – Fitted parameters from electrochemical impedance spectroscopy for the three different 3D printed flow fields with the Freudenberg carbon paper electrode at 0.5 cm $s^{-1}$ electrolyte velocity.

| Flow field $v_e = 0.5$ cm $s^{-1}$ | $R_{TOT}$ [Ω $cm^2$] | $R_{ohm}$ [Ω $cm^2$] | $R_{CT}$ [Ω $cm^2$] | $R_{MT}$ [Ω $cm^2$] |
|---|---|---|---|---|
| ID | 2.593 | 1.739 | 0.291 | 0.564 |
| Lung 2L | 2.058 | 1.387 | 0.117 | 0.553 |
| Lung 3L | 2.068 | 1.377 | 0.196 | 0.495 |

**Table S9** – Fitted parameters from electrochemical impedance spectroscopy for the three different 3D printed flow fields with the Freudenberg carbon paper electrode at 1.5 cm $s^{-1}$ electrolyte velocity.

| **Flow field** $v_e = 1.5\ \mathrm{cm\ s^{-1}}$ | $R_{TOT}$ **[Ω cm²]** | $R_{ohm}$ **[Ω cm²]** | $R_{CT}$ **[Ω cm²]** | $R_{MT}$ **[Ω cm²]** |
|---|---|---|---|---|
| ID | 2.109 | 1.683 | 0.196 | 0.230 |
| Lung 2L | 1.711 | 1.336 | 0.156 | 0.219 |
| Lung 3L | 1.760 | 1.392 | 0.156 | 0.212 |

**Table S10** – Fitted parameters from electrochemical impedance spectroscopy for the three different 3D printed flow fields with the Freudenberg carbon paper electrode at 3.5 cm $s^{-1}$ electrolyte velocity.

| **Flow field** $v_e = 3.5\ \mathrm{cm\ s^{-1}}$ | $R_{TOT}$ **[Ω cm²]** | $R_{ohm}$ **[Ω cm²]** | $R_{CT}$ **[Ω cm²]** | $R_{MT}$ **[Ω cm²]** |
|---|---|---|---|---|
| ID | 2.007 | 1.691 | 0.143 | 0.173 |
| Lung 2L | 1.517 | 1.295 | 0.120 | 0.102 |
| Lung 3L | 1.632 | 1.392 | 0.140 | 0.099 |

**Table S11** – Fitted parameters from electrochemical impedance spectroscopy for the three different 3D printed flow fields with the Freudenberg carbon paper electrode at 5 cm $s^{-1}$ electrolyte velocity.

| **Flow field** $v_e = 5\ \mathrm{cm\ s^{-1}}$ | $R_{TOT}$ **[Ω cm²]** | $R_{ohm}$ **[Ω cm²]** | $R_{CT}$ **[Ω cm²]** | $R_{MT}$ **[Ω cm²]** |
|---|---|---|---|---|
| ID | 1.989 | 1.721 | 0.130 | 0.138 |
| Lung 2L | 1.474 | 1.267 | 0.133 | 0.074 |
| Lung 3L | 1.614 | 1.377 | 0.168 | 0.069 |

## References


(1) Sadeghi, M. A.; Aganou, M.; Kok, M.; Aghighi, M.; Merle, G.; Barralet, J.; Gostick, J. Exploring the Impact of Electrode Microstructure on Redox Flow Battery Performance Using a Multiphysics Pore Network Model. *J. Electrochem. Soc.* **2019**, *166* (10), A2121–A2130. DOI 10.1149/2.0721910jes.

(2) Nafion 212 Datasheet. DuPont™ Nafion® PFSA Membranes.

(3) Aziz, Md. A.; Shanmugam, S. Sulfonated Graphene Oxide-Decorated Block Copolymer as a Proton-Exchange Membrane: Improving the Ion Selectivity for All-Vanadium Redox Flow Batteries. *J. Mater. Chem. A* **2018**, *6* (36), 17740–17750. DOI 10.1039/C8TA06717A.

(4) Forner-Cuenca, A.; Penn, E. E.; Oliveira, A. M.; Brushett, F. R. Exploring the Role of Electrode Microstructure on the Performance of Non-Aqueous Redox Flow Batteries. *J. Electrochem. Soc.* **2019**, *166* (10), A2230–A2241. DOI 10.1149/2.0611910jes.

(5) Greco, K. V.; Forner-Cuenca, A.; Mularczyk, A.; Eller, J.; Brushett, F. R. Elucidating the Nuanced Effects of Thermal Pretreatment on Carbon Paper Electrodes for Vanadium Redox Flow Batteries. *ACS Appl. Mater. Interfaces* **2018**, *10* (51), 44430–44442. DOI 10.1021/acsami.8b15793.

(6) Sun, C.-N.; Delnick, F. M.; Baggetto, L.; Veith, G. M.; Zawodzinski, T. A. Hydrogen Evolution at the Negative Electrode of the All-Vanadium Redox Flow Batteries. *J. Power Sources* **2014**, *248*, 560–564. DOI 10.1016/j.jpowsour.2013.09.125.

(7) van der Heijden, M.; Forner-Cuenca, A. Transport Phenomena and Cell Overpotentials in Redox Flow Batteries; p 20.
